# Micro‐CT guided illustration of the head anatomy of penguins (Aves: Sphenisciformes: Spheniscidae)

**DOI:** 10.1002/jmor.21476

**Published:** 2022-04-14

**Authors:** Peter W. Hadden, William C. Ober, Dane A. Gerneke, Daniel Thomas, Miriam Scadeng, Charles N. J. McGhee, Jie Zhang

**Affiliations:** ^1^ Department of Ophthalmology, New Zealand National Eye Centre, Faculty of Medical and Health Sciences University of Auckland Auckland New Zealand; ^2^ Shoals Marine Laboratory Cornell University Ithaca New York USA; ^3^ Shoals Marine Laboratory University of New Hampshire Durham New Hampshire USA; ^4^ Auckland Bioengineering Institute University of Auckland Auckland New Zealand; ^5^ School of Natural Sciences Massey University Auckland New Zealand; ^6^ Department of Academic Radiology, Faculty of Medical and Health Sciences University of Auckland Auckland New Zealand

**Keywords:** avian orbit, bird head, bird skull, jaw muscles, penguin

## Abstract

The illustration is an important tool to aid in the description and understanding of anatomy, and penguins (Aves: Sphenisciformes: Spheniscidae) are an important clade in environmental monitoring, paleontology, and other research fields. Traditionally, anatomic illustration has been informed by dissection. More recently, micro‐computed tomography (micro‐CT) has proven to be a powerful tool for three‐dimensional anatomic imaging, although larger specimens are more challenging to image due to increased X‐ray attenuation. Here, we used traditional dissection and micro‐CT to illustrate the skulls of *Aptenodytes patagonicus*, *Eudyptula minor*, and *Pygoscelis papua*, and the extracranial soft tissue of *E. minor*. Micro‐CT prevented the loss of orientation, disarticulation, and distortion of bones that might result from cleaning and drying skulls, while immobilization was achieved by freezing the specimens before imaging. All bony elements in the head were accurately depicted. Fixing, dehydrating, and diffusion staining with iodine (diceCT) enabled the identification of muscles and other large nonmineralized structures, but specimen preparation precluded the ability to show smaller nerves and vessels. The results presented here provide a guide for anatomic studies of penguins and our summary of sample preparation and imaging techniques are applicable for studies of other similarly sized biological specimens.

## Graphical abstract

This illustration is a composite left lateral view of the deeper muscles of the head of a little penguin (*Eudyptula minor*) and a micro‐computed tomography image of the skull from which the bony elements were drawn, demonstrating the usefulness of traditional illustration, informed by more modern methods as well as dissection, in understanding the anatomy of the penguin head.

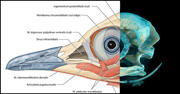

## 1 INTRODUCTION

Penguins (Aves: Sphenisciformes: Spheniscidae) are an important clade in multiple research disciplines including, for example, animal physiology, coastal ecosystem health, and paleontology. Extant species of Spheniscidae have been the subject of particular anatomic attention, being the extant representatives of an ancient lineage with a fossil record as old as the early Paleocene and distinct from other birds for the entirety of the Cenozoic (Jadwiszczak, 2009; Ksepka & Ando, 2011; Slack et al., 2006; Triche, 2007). Although other avian lineages share some of the features of Spheniscidae, including a long history of flightlessness (e.g., ratites such as the emu, *Dromaius novaehollandiae*) and diving ability (e.g., the common murre common guillemot, *Uria aalge*, Piatt & Nettleship, 1985), penguins are the only surviving lineage of flightless, wing‐propelled diving birds (Tambussi et al., 2015).

The illustration is an important tool to aid in the understanding of anatomy and numerous illustrations of avian head anatomy are readily available (Baumel et al., 1993; Ghetie et al., 1976; Sosa & Acosta Hospitaleche, 2018). Anatomic studies of the penguin head include descriptions of the skull and the muscles of the jaw (Zusi, 1975), the supraorbital gland of *Pygoscelis* penguins (Herbert, 1975), the skull and musculature of the emperor penguin (Sosa & Acosta Hospitaleche, 2018) and the skulls of *Pygoscelis* penguins (Acosta Hospitaleche & Tambussi, 2006). Detailed comparative anatomic descriptions of the head are also available for both extinct and extant penguins (Degrange et al., 2018; Tambussi et al., 2015; Triche, 2007). However, neither illustrated anatomic diagrams of the head comparable to those found in human anatomic textbooks nor micro‐computed tomography (micro‐CT) renders of the head of the penguin are available, despite their usefulness for didactic purposes or in the veterinary care of these animals.

Micro‐CT has emerged as a powerful imaging tool for natural history specimens and comprehensive head anatomy of birds including the dove (*Columba livia*; Jones et al., 2019) and common buzzard (*Buteo buteo*; Lautenschlager et al., 2014) as imaged by micro‐CT has been published. Bone can be particularly well imaged with this technique, although care has to be taken to prevent movement during the scanning procedure. The use of X‐ray contrast media (contrast‐enhanced CT [CE‐CT]) aids the visualization of nonmineralized tissue (Metscher, 2009a) and can allow histological‐scale resolution of embryonic tissue (Metscher, 2009b). Diffusible iodine‐based contrast‐enhanced computed tomography (diceCT) has become increasingly popular due to its low cost, ease of handling, and its ability to differentiate between major types of soft tissue (Gignac et al., 2016). The penguin's head is larger than that of both the dove and common buzzard. However, similarly sized soft tissue structures from other animals have been successfully imaged using diceCT (Gignac & Kley, 2014; Gignac et al., 2016; Lupon et al., 2020).

Here, our aim was to develop easily interpretable illustrations of the extracranial anatomy of the penguin head, equal in quality to those seen in human anatomy texts, to support teaching, and research and to aid veterinarians in the interpretation of diagnostic imaging and when undertaking therapeutic interventions. We concentrated on the bones and large organs, excluding the illustration of Systema Nervosa and Systema Cardiovasculare, both because of the condition in which the specimens arrived and for the sake of brevity. We have also included in the associated repository reconstructions from the micro‐CT images, including files that can be 3D‐printed for didactic purposes. By developing these resources for penguins, we also aimed to show the utility both of anatomic illustration as a facilitator in the understanding of anatomy and of micro‐CT for resolving fine anatomic features, and thereby encourage the development of similar resources for other important research taxa.

We elected to use the head of a little penguin (*Eudyptula minor*) for this purpose. This animal is known in *te reo Māori* as kororā and we have used these common names interchangeably. We also examined the heads of both gentoo (*Pygoscelis papua*) and king (*Aptenodytes patagonicus*) penguins, concentrating on the skeletal elements. We anticipated that the highest resolution images would be obtained from the little penguin, given the smaller tissue volume and the scanner able to be used for this head, and this influenced our decision to concentrate on that species. Although primarily the reason for selecting these species was their availability in Auckland, New Zealand, nevertheless they include both the smallest penguin, the kororā, which rarely dives below 60 m (Montague, 1985), as well as the second‐largest extant penguin, the king, capable of diving to over 300 m (Culik et al., 1996). They also occupy a diverse range of habitats, from warm temperate shores to subantarctic islands (Shirihai, 2007), and represent different lineages within Spheniscidae (Baker et al., 2006; Zusi, 1975), with recent DNA evidence suggesting that *Aptenodyte*s, perhaps along with *Pygoscelis*, are sister to all other extant penguins (Cole et al., 2019; Vianna et al., 2020). We aimed, therefore, to uncover novel anatomic features of Spheniscidae and perhaps differences between genera, apart from the obvious difference in size.

## 2 MATERIALS AND METHODS

### 2.1 Specimens

We obtained the heads of three little penguins (kororā), *Eudyptula minor* (Forster, 1781) (L1, L2, and L3), two gentoo penguins, *Pygoscelis papua* (Forster, 1781) (G1 and G2) and four *king penguins* (Miller, 1778; K1, K2, K3, and K4). All kororā were adults, had been found dead of natural causes in coastal areas of the Auckland Region, New Zealand and had been stored frozen before our obtaining them. The gentoo and king specimens had been bred in captivity and were resident in SEA LIFE Kelly Tarlton's Aquarium in Auckland but were of South Georgian descent and had been euthanized either because of age‐related functional deterioration or, in the case of G2, failure to thrive at 7 weeks old. G2 was thus the only juvenile bird in the series. Only the heads of L1, L2, G1, and K1 were completely intact when we received them, the others having been subject to postmortem examination or, in the case of L3, cleaned by cold water maceration over a period of months. What remains of these now mostly skeletal specimens are held in the Department of Ophthalmology, Faculty of Medical and Health Sciences, University of Auckland. The eye of one little penguin in the care of Auckland Zoo was filmed to ascertain pupil shape and color under both mesopic and photopic conditions, while intubated under general anesthesia for the purposes of having its beak trimmed. All specimens were obtained and examined with permission from the New Zealand Department of Conservation (68003‐DOA) and ethics approval from SEA LIFE Kelly Tarlton's Aquarium (SL[G] AR001) and Auckland Zoo. All raw data, including micro‐CT, photographs, and the pupil video, are available in the online open access repository https://figshare.com/search?q=10.17608/k6.auckland.c.5599341 (Digital Science).

### 2.2 Specimen preparation and imaging

Because stabilization of large, unfixed specimens during a long acquisition period is challenging, particularly when imaging in air, and because the heads were frozen when we received them, we imaged all specimens frozen, as previously described (De Rycke et al., 2012; Ferrare et al., 2013; Green & Gignac, 2021). Micro‐CT data acquisitions of the little penguin head were conducted using a Bruker Skyscan 1172 while for the gentoo head we used a Bruker Skyscan 1272. The most caudal part of the head of G1 and beaks of both L1 and G1 were removed from the rest of the head and their feathers shortened with scissors to allow them to fit into the micro‐CT scanner, as the 1172 instrument (used for L1) can only fit a cylinder up to 50 mm in diameter and 70 high, while the maximum volume that can be scanned with the 1272 instrument (used for G2) is a cylinder 75 mm in diameter and 80 mm high, although removal of the plate can allow a sample of 100 mm to be placed in the instrument. The large volume of the head ensured that a complete thaw did not occur during scanning, thus preventing movement artefact. The images were reconstructed using InstaRecon CBR Server Premium 15K (InstaRecon Inc.). Analysis was done using CTAnal V1.18.4.0 (Bruker) and additional editing was done using Adobe Photoshop CC V19.1.3 (RRID:SCR_014199). Data from the king penguin head was acquired with an X25 CT system NSI (North Star Imaging). The beak of K1 did not fit into the field that was imaged but did not require shortening to fit into that more open industrial scanner. The software used for analysis was Geomagic Design X (Geomagic Inc.). Further details on scanning parameters are presented in Table 1. All data were subsequently visualized in three dimensions using CTVox V 3.3 (Bruker) and Amira 2021.2 (Thermo Fisher Scientific). Data View V 1.5.4.0 (Bruker) was used to adjust rotational and tilt orientation to give comparable views of all samples. As it provides two‐dimensional transverse, coronal and sagittal planes with accurate dimensions, Data View V 1.5.4.0 was also used to obtain measurements. Three‐dimensional visualization programs such as CTVox are not as accurate in this regard because of distortions induced by perspective, a function of the camera in that program (Hadden et al., 2021).

**Table 1:** The imaging conditions used for micro‐CT of the penguin heads

Sample	CT	kV	µA	Filter Al.	Exposure time (ms)	µm pixel resolution	Rotation step (deg.)	Rotation (deg.)	Number of fields	Scan time (hours)	Frame Ave.	Random movement
Little	1172	100	100	1.0	1100	27.09	0.5	360	6	6.25	2	6
Gentoo	1272	100	100	0.5	1500	20.0	0.6	360	18	15.5	2	6
King	X25	150	100	None	‐	170.8	Helical	360 × 12	‐	0.25	‐	‐

After enucleation of Bulbus oculi dextri, the head of L1 was defrosted and fixed in 1% formaldehyde and 1.25% glutaraldehyde, both to improve the penetration by the contrast agent and allow visualization in air, the latter providing good contrast with a tissue while reducing the density of the specimens to X‐ray. The specimen was then dehydrated using increasing concentrations of ethanol over 18 days, to remove lipid content and this reduction in tissue volume further reduces the energy required while imaging (Lupon et al., 2020). It was then diffusion stained with alcoholic potassium triiodide (IKI) solution 0.75% for a period of 14 days before being briefly washed in 70% alcohol before rescanning using the same Bruker Skyscan 1172 and software as described above.

To confirm the anatomy obtained through imaging and to answer remaining uncertainties, following micro‐CT the heads of L1 and G1 were dissected, L1 having been fixed and stained as described above while G1 was defrosted and dissected fresh. The heads of L2, G2, K3, and K4 were also dissected fresh while that of K2 had been preserved by means of immersion in a 4% formaldehyde solution for 3 months. Dissection was performed both under direct visualization and, to identify finer details, using a Lumera 700 ceiling‐mounted operating microscope (Carl Zeiss AG). Photographs were taken during dissection for purposes of documentation. Soft tissue was subsequently removed from the skulls of G1, G2, and K4 by means of burial for 2 months in soil, to allow osteological examination. Two photographs were taken of the lateral aspect of the skull of L3, one straight lateral and one from an angle of 30° dorsally and 30° caudally, to create shadowing for the purpose of accurate illustration. Two photographs were also taken of the ventral aspect, one straight ventral and one from an angle of 30° laterally and 30° caudally. Additional editing of photographs was accomplished using Adobe Photoshop CC V19.1.3 (RRID:SCR_014199).

**Figure 1:**
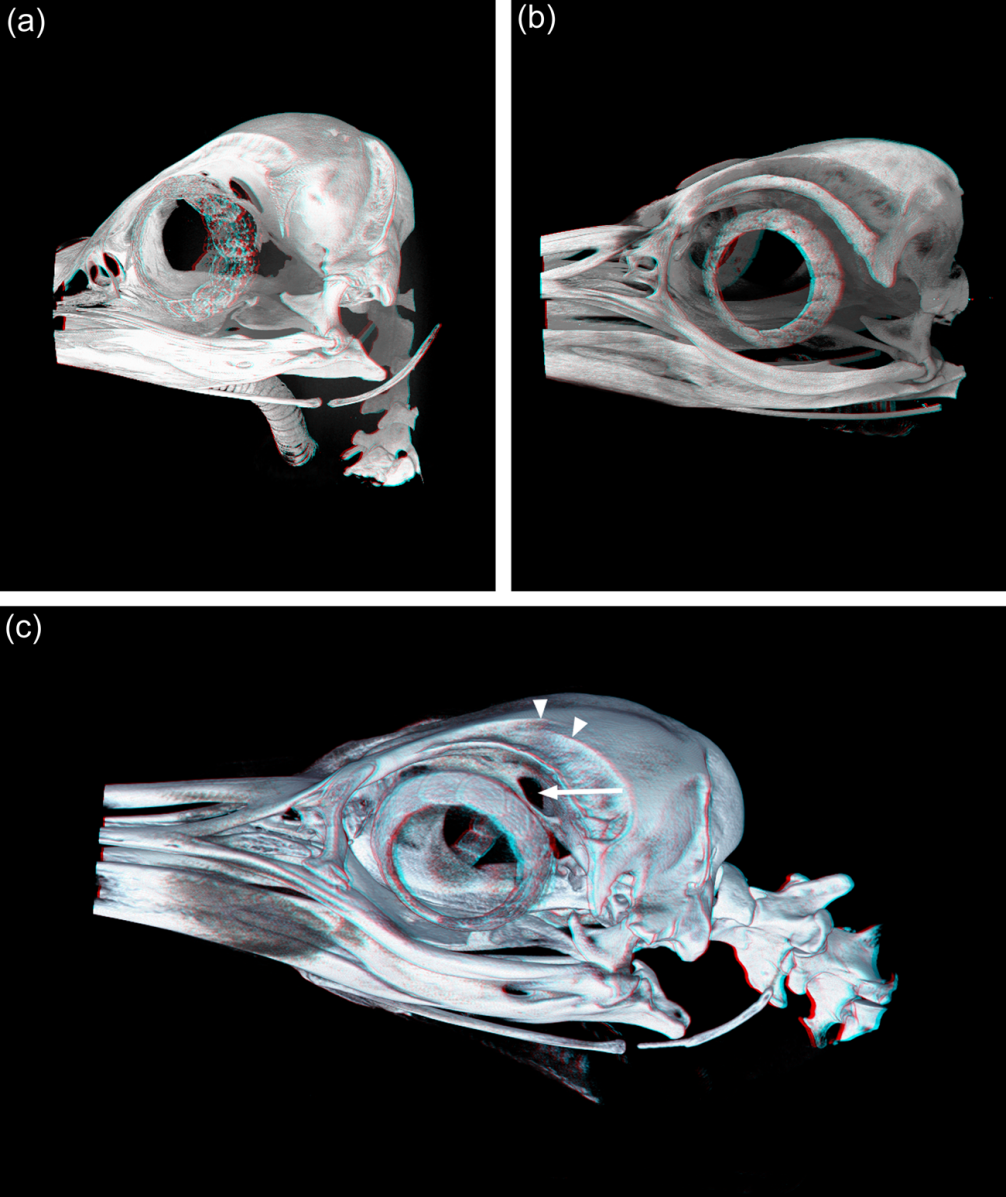
Three‐dimensional CTVox reconstructions of frozen head micro‐CT scans. (a) Little penguin (*Eudyptula minor*) L1. (b) Gentoo penguin (*Pygoscelis papua*) G1. (c) King penguin (*Aptenodytes patagonicus*) K1. (a) and (b) are approximately left lateral views. The view of the king penguin (c) is of the right side of the skull and has been rotated 180° around the *y* axis for ease of comparison with the other figures. It has also been rotated 10° around the *x* axis to display a foramen in Paries caudalis orbitae (arrow). These images may be viewed stereoscopically using red‐green glasses. The detail visible in K1 is markedly lower than that visible in L1 or G1 due to the lower resolution scanner required to image the larger head. There is also an artificial discontinuity present at the level of the mid orbit of K1, due to transient vibration during the imaging process in a factory environment (the arrowheads mark the medial border of Fossa glandulae nasalis, which should be continuous)

A ghost, or artifactual bony discontinuity, was observed in the transaxial planes of the image of K1, in the region of Fossa glandulae nasalis and Os jugale (Figure 1). We initially suspected that this was due to transient vibration of the scanner during acquisition, as it is situated in a factory environment with machine shops in fairly close proximity and is not in the same class of instrument as the other scanners. However, more likely was a timing error, whereby the next scan field start is delayed momentarily, resulting in displacement. The software fuses the transaxial planes by fading out the top of the bottom and fading in the bottom of the one above. We were unable to rescan the head in the same machine because of Covid‐19 related restrictions preventing our entry to the factory in which this machine was located. Therefore, to ensure that this appearance was artefactual, we reimaged this head using a Siemens SOMATOM Perspective CT scanner (Siemens Medical Solutions USA, Inc.), although the resolution afforded by this scanner was lower. We attempted to correct the micro‐CT using Amira and by the use of *x*/*y* alignment algorithms but this was not successful as the ghosting was rotational and offset from the strong bone signal. Manually erasing the ghost areas in the affected transaxial planes using Photoshop Creative Cloud was also not successful due to thresholding. Hence no.stl file of K1 was able to be created.

### 2.3 Scientific illustration

The illustrator, WCO, was unable to view any specimen in person and therefore had to draw the skulls of specimens L1, G1, and K1 using the micro‐CT data as the primary source and, secondarily, the aforementioned skull photographs. As they were not imaged, the beaks of all three penguins were illustrated only using photographs of the skull, as was Epibranchiale of G1. All initial drafts were reviewed by PWH, who had access to the primary specimens and was able to make micro‐CT renders from the raw data as required. PWH then made sketches of the modifications necessary to ensure accuracy, attaching relevant micro‐CT renders and macro photographs as appropriate. This process was repeated until both were satisfied with the illustration produced. Following the completion of accurate illustrations of the skull of L1, the large soft tissue organs, namely the muscles, exocrine glands (glandulae exocrinae) and Bulbus oculi were then sketched onto the illustrations of the bony anatomy of the little penguin by PWH, using a combination of information gathered through diceCT and dissection. WCO then illustrated these sketches and these were further modified by PWH in the same manner as was done for the skulls.

Proper specimen preparation is essential to useful imaging. All our specimens had been frozen while K2, K3, K4, and G2 had also been partially dissected for postmortem examination. The head of L1 was also further treated as above to enhance micro‐CT discrimination between soft tissue structures. This disruption of the vasculature these steps entailed precluded the use of perfusion staining of the vasculature. Further, neural tissue is known to be particularly sensitive to disruption by freezing and the removal of lipid during the processing, to which iodine appears to bind, is known to reduce contrast between myelinated nerves and lipid‐poor tissues (Gignac & Kley, 2018; Gignac et al., 2016). Hence, Systema Cardiovasculare and Systema Nervosa were unable to be imaged.

Anatomic features were labeled according to the Nomina Anatomica Avium (NAA) (Baumel et al., 1993). Holliday and Witmer (2007) have made more recent suggestions regarding the labeling of the muscles of the adductor chamber and for this reason, we labeled these muscles with both. The NAA notes various subdivisions of Musculus pterygoideus have been described in different avian species. Zusi (1975) described this muscle in the kororā and found the same subdivisions as did we, although he altered the names he had given to these subdivisions in a subsequent paper describing the same muscle and the same subdivisions in hummingbirds (Aves: Trochlilidae; Zusi & Bentz, 1984). Given that we were also examining the species and that our findings were identical, we used his later nomenclature for these subdivisions.

## 3 RESULTS

### 3.1 Micro‐CT images and illustrations

**Figure 2:**
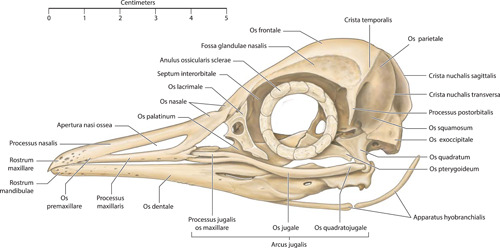
*Eudyptula minor*, illustration of the skull (specimen L1), left lateral view

**Figure 3:**
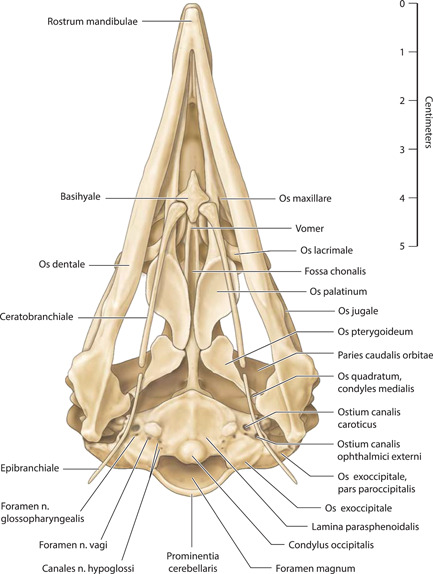
*Eudyptula minor*, illustration of the skull (specimen L1), ventral view

**Figure 4:**
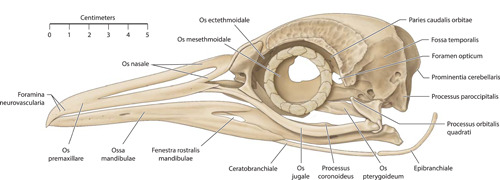
*Pygoscelis papua*, left lateral view of the skull (specimen G1). Examination of the 7‐week‐old G2 revealed a foramen in Paries caudalis orbitae not present in G1. Epibranchiale was not visible in the micro‐computed tomography image and was drawn solely using cleaned bone and estimating its position from dissection

**Figure 5:**
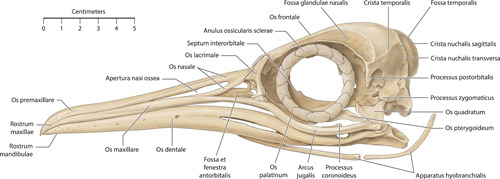
*Aptenodytes patagonicus*, illustration of the skull (specimen K1). Left lateral view. In three king penguins, K1, K2, and K4, there was a round nonossified foramen in the center of Paries caudalis orbitae (not visible from this angle but shown on Figure 1). In king penguin K3, this area was still visible as a thin but clearly calcified area on dissection

**Figure 6:**
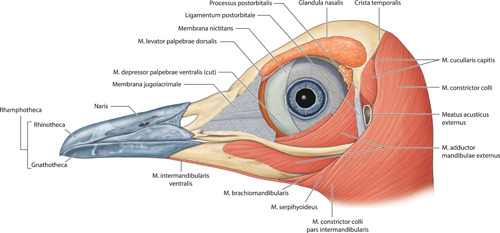
*Eudyptula minor*, illustration of the most superficial muscles of the head (specimen L1), left lateral view. Partes ventralis et profundus of Musculus adductor mandibulae externus were inseparable at their wide insertion on the external surface of ossa mandibulae. Musculus levator palpebrae dorsalis and Musculus orbicularis oculi (not pictured) were virtually impossible to see with the naked eye, but just visible on micro‐computed tomography

**Figure 7:**
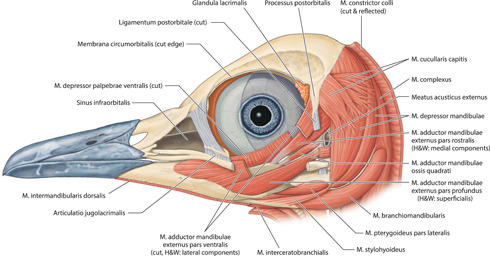
*Eudyptula minor*, left lateral view of the deeper muscles of the head (specimen L1). While Musculus cucullaris capitis is depicted as having two major divisions in this illustration, in L2 only the equivalent of the more ventral division was present (Appendix 10). Musculus depressor mandibulae appeared to have two divisions, one larger and more rostral to the other. Where anatomic nomenclature varies between Nomina Anatomica Avium (Baumel et al., 1993) and Holliday and Witmer (2007), the former has been used and the latter noted

**Figure 8:**
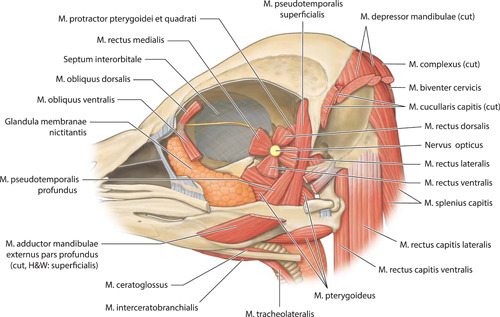
*Eudyptula minor*, drawing of the deepest extracranial structures visible from the side in the head (specimen L1). Left lateral view. Where anatomic nomenclature varies between Holliday and Witmer (2007) and Nomina Anatomica Avium (Baumel et al., 1993), the latter has been used and the former noted

**Figure 9:**
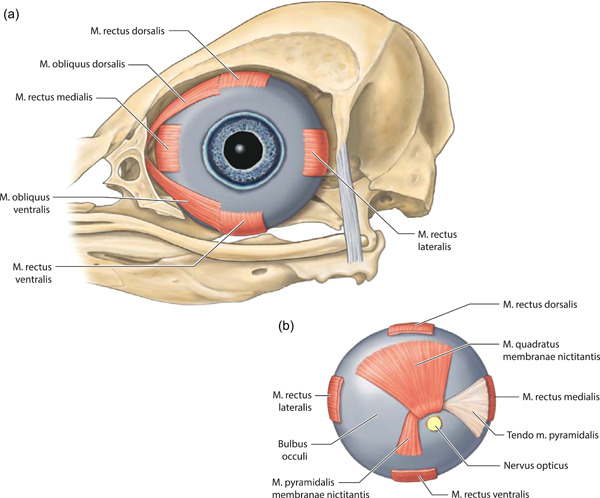
*Eudyptula minor*, Bulbus oculi sinistri and Musculi bulbi (specimen L1). The pupil was drawn based on our observation of a different, living kororā. When the pupil was the size drawn here it appeared slightly octagonal rather than completely round, although when dilated it appeared circular. The axial length of the globe was 14 mm and the equatorial diameter was 19 mm, as measured on micro‐computed tomography (a) Left lateral view. (b) A posterior view of Bulbus oculi sinistri

**Figure 10:**
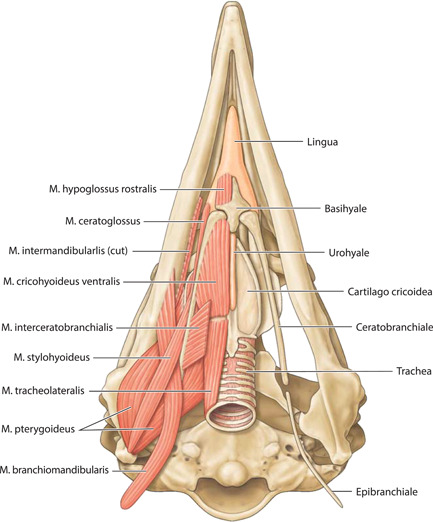
*Eudyptula minor*, Apparatus hyobranchialis and associated structures (specimen L1), ventral view. With the exception of Urohyale, the laryngeal skeleton was clearly ossified on micro‐computed tomography

**Figure 11:**
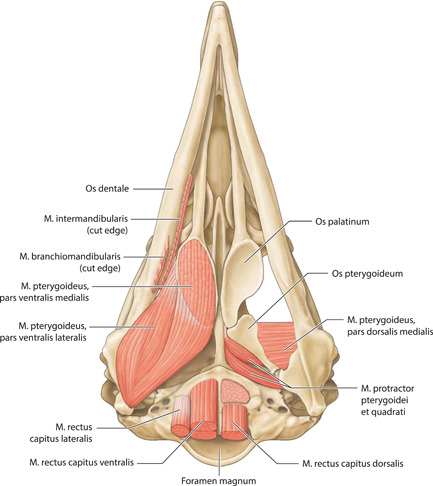
*Eudyptula minor*, illustration of the cranial musculature (specimen L1), ventral view

Micro‐CT skeletal reconstructions of transaxial scans of each frozen head (L1, G1, and K1) made using the.bmp raw data are presented in Figure 1; a stereoscopic image may be obtained by using red‐green glasses. More CTVox (freeware that allows three‐dimensional visualization) reconstructions made using this same data, both stereoscopic and nonstereoscopic, of all three skulls, the transfer functions used to create these reconstructions, .stl files of L1 and G1 suitable for 3D printing and the DICOM files from the Siemens CT performed on K1 due to the observed artefact on the X25 CT system NSI scanner are available at https://figshare.com/search?q=10.17608/k6.auckland.c.5599341, the same repository that contains the raw data referred to earlier. The illustrations produced from these images, in combination with cleaned skulls as described above, are presented in Figures 2, 3, 4, 5. The same figshare also contains thick coronal sections reconstructed using CTVox from the diceCT of L1, the raw data of the diceCT reconstructed and exported as 8 bit.bmp or 16 bit.tif files, CTVox renderings made using either file type, and Amira 2021.2 (.am) files with the major skeletal elements, muscle groups and other solid organs labeled and segmented. The illustrations of the nonmineralized extracranial tissues of L1 produced using these images, aided by dissection, are presented in Figures 6, 7, 8, 9, 10, 11.

### 3.2 Skeletal elements

Basic data regarding skull dimensions is presented in Table 2. There were clear differences in size, which correlated with the size of the animal.

**Table 2:** Selected skull measurements

Sample	Total length of skull (mm)^a^	Skull width (mm)^b^	Skull depth (mm)^b^	Salt gland fossae (mm)^b^	Length of bill (mm)^a^	Width at base of bill (mm)^b^	Width of internarial bar (mm)^a^	Distance between pupils (mm)^b^
Little (*Eudyptula minor)*	103	35.0	28.0	4.7	40	11.1	3.5	31
Gentoo (*Pygoscelis papua*)	172	51.8	45.0	3.1	78	21.8	3.2	51
King (*Aptenodytes patagonicus)*	212	55.3	71.4	12	105	20.0	4.7	89

*Note*: The interpupillary distance was measured between the center of each bony pupil, as determined by the center of the anterior aperture of Anulus ossicularis sclerae on micro‐CT.

^a^Measured directly from the skull.

^b^Measured using data view.

**Figure 12:**
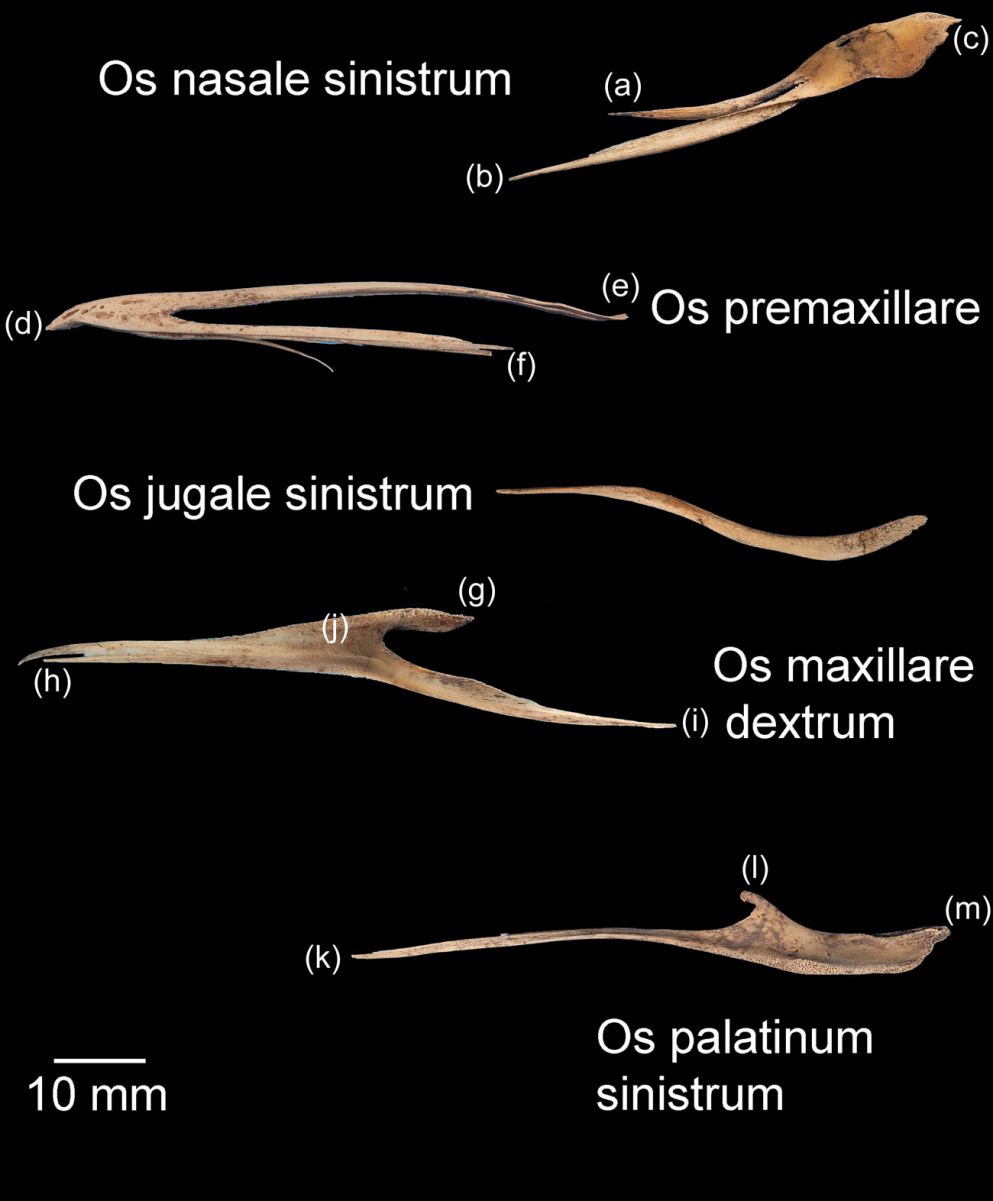
*Pygoscelis papua*, macrophotographs of the disarticulated bones of Maxilla and the small bones with which it articulates caudally (specimen G2). The rostral portion of Maxilla was unable to be imaged in any penguin and thus we were reliant on cleaned specimens for illustration; however, these are subject to distortion by cleaning (burial in soil). Os nasale sinistrum, left lateral view: (a) Processus premaxillaris. (b) Processus maxillaris. (c) Processus frontalis. Os premaxillare (an unpaired bone), left lateral view: (d) Rostrum maxillae. (e) Processus frontalis. (f) Processus maxillaris. Note the distortion of Processus frontalis and Processus maxillaris caused by cleaning; in intact specimens both are straight. Os jugale sinistrum, left lateral view: We could not identify Os quadratojugale in this specimen nor on micro‐CT in G1. Os maxillare dextrum, ventral view: (g) Processus maxillopalatinus, (h) Processus premaxillaris, (i) Processus jugalis, (j) The groove in which Processus maxillaris ossis palatini sits. Os palatinum sinistrum, left lateral view: (k) Processus maxillaris (syn. Processus premaxillaris), (l) Processus rostralis, and (m) Processus pterygoideus

The rostral portion of Maxilla was unable to be imaged by micro‐CT in any animal but disarticulated with ease in the juvenile gentoo penguin G2 (Figure 12). Its tip, Rostrum maxillae, was formed by Os premaxillare and featured a ventral curve. Os premaxillare was penetrated by numerous Foramina neurovascularia on each side and articulated caudally via long, slender processes with the more caudal skeletal elements. These included Ossa nasalia dorsally, Ossa maxillaria ventrolaterally and Ossa palatina ventromedially. Processus maxillaris ossis palatini (synonymous with Processus premaxillaris ossis palatini) fitted into a groove on the ventral surface of Os maxillare between the base of Processus maxillopalatinus ossis maxillaris and Processus jugalis ossis maxiallaris. Together these bones formed the composite Maxilla, with the extended contact zones between each skeletal element, the Zonae flexoriae, allowing some bending (Baumel et al., 1993). Close to Calvaria, each side of Maxilla diverged from the other at an angle of 15° in all penguins, but they became more parallel rostrally such that Maxilla ended in a tapering tip, being more elongated relative to body size in the king penguin than in the other species (compare Figures 2, 4, and 5). The lateral aspect of Maxilla was pierced by Apertura nasi ossea while the dorsal surface sloped gradually upwards as it proceeded caudally and the ventral surface had a sharp lateral edge, Crista tomialis.

The prominent vault of Cranium (more specifically, Neurocranium) was formed by Calvaria which, together with Basis cranii externa, enclosed Encephalon (Figure 12). As in other adult birds, Cranium was formed from the fusion of the convex Ossa frontalia together with Os basioccipitale, Ossa exoccipitalia, Os supraoccipitale, Ossa laterosphenoidalia, Os basisphenoidale, Os parasphenoidale, Ossa squamosa, Ossa parietalia, Os mesethmoidale and Ossa ectethmoidalia. We were unable to visualize Sutura cranii between these bones on either micro‐CT or direct inspection of the skull. Rostrally the convex Ossa frontalia descended to articulate with Ossa nasalia. Extending along Margo supraorbitalis from Sutura lacrimofrontalis to Processus postorbitalis, widening caudally, was the fenestrated Fossa glandula nasalis (Figure 1). In both the king and gentoo penguin there was a prominent ridge on its lateral margin, although the fossa was deeper and the ridge more developed in the gentoo such that Glandula nasalis fitted snugly into the fossa. In contrast, in the little penguin this ridge was absent and Glandula nasalis overhung Orbita rather than being fully contained within a bony fossa. Caudally this fossa was terminated by Crista temporalis, a feature which also delimited the rostral extent of Fossa temporalis. This latter fossa extended caudally, becoming deeper as it did so, before terminating at the sharp edge of Crista nuchalis transversa, a ridge which extended toward but did not cross the sagittal line dorsally. The quadrangular occipital aspect of Calvaria was dominated centrally by Prominentia cerebelli, its most caudal point, dorsal to which was the midline Crista nuchalis sagittalis. Ventrally, Basis cranii externa was dominated by the circular Foramen magnum, where Cranium articulated with Atlas at Articulatio atlanto‐occipitalis and through which passed Medulla spinalis and Meninges as they headed caudally through Columna vertebralis. Immediately rostral to Foramen magnum was Condylus occipitalis while to each side were multiple ostia and canales for nervi craniales, Arteria carotis cerebralis and Vena carotis cerebralis.

**Figure 13:**
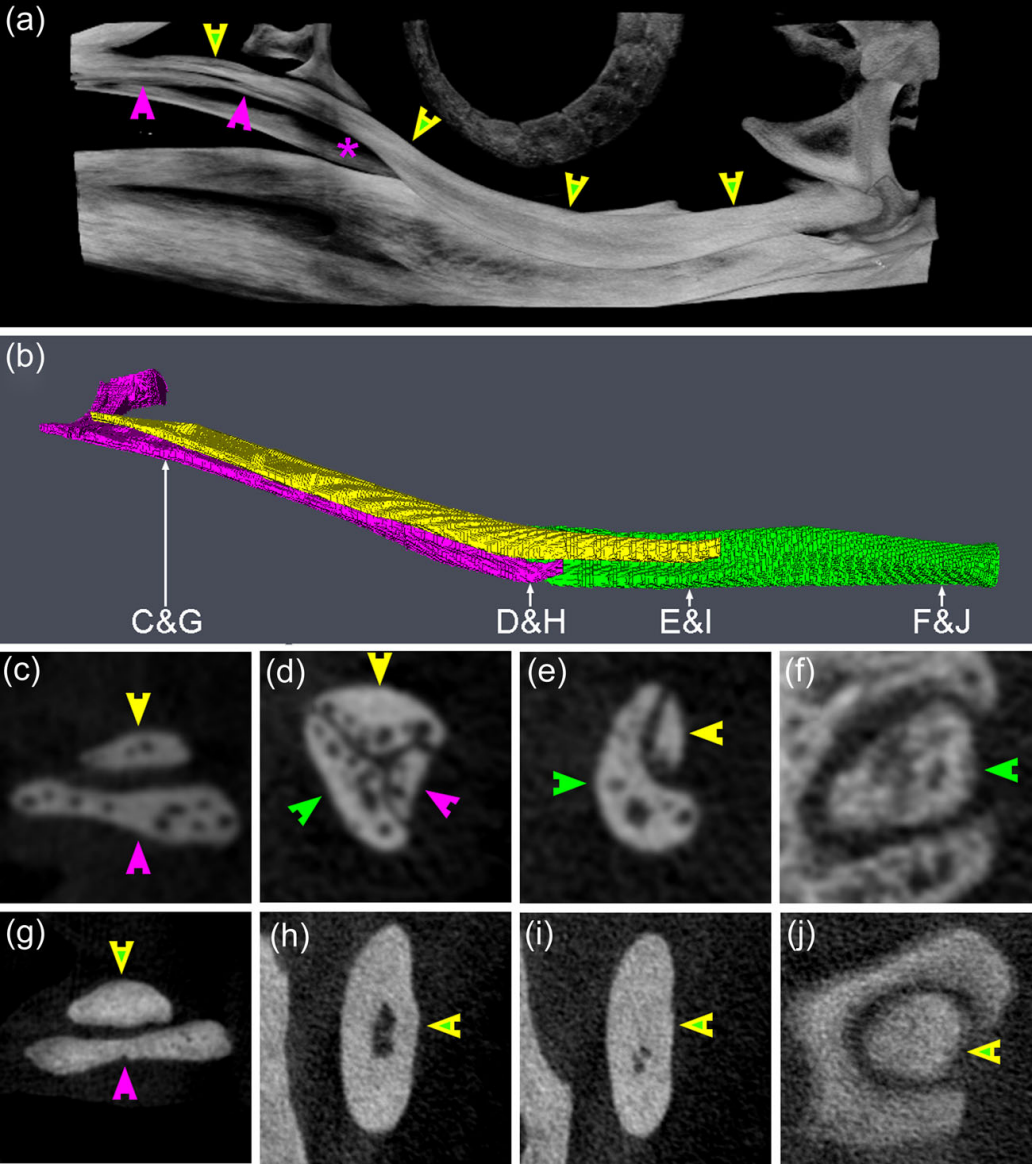
*Pygoscelis papua*, micro‐CT reconstructions of Arcus jugalis (specimen G1). (a) CTVox reconstruction of Arcus jugalis sinister. Purple arrowheads indicate Processus jugalis ossis maxillaris, with the purple asterix at its caudal termination, while green and yellow arrowheads indicate Os jugale and Os quadratojugale, indistinguishable in this penguin. (b) Amira 6.5.0 reconstruction of Arcus jugalis sinister in little penguin (*Eudyptula minor*) L1. Processus jugalis ossis maxillaris is colored purple, Os jugale yellow and Os quadratojugale green. (c)–(f) Coronal micro‐CT sections through Arcus jugalis sinister of L1 at the points indicated on (b). Note that Processus jugalis ossis maxillaris (purple arrowheads), Os jugale (yellow arrowheads) and Os quadratojugale (green arrowheads) are able to be separately identified in this penguin, all three being present in section D. (g)–(j) Coronal micro‐CT images through Arcus jugalis sinister of G1 at approximately equivalent locations to those in L1. Unlike L1, however, at no point was Os jugale able to be separated from Os quadratojugale in G1. Because Processus jugalis ossis maxillaris did not extend as far caudally in G1 as in L1, it is not present in section H

The paired Orbitae were partially separated by Os mesethmoidale, part of the fused Calvaria, which formed most of the rostral, osseous portion of Septum interorbitale. There was significant osteological variation within birds of the same species in Paries caudalis orbitae; the orbital aspect of Os laterosphenoidale in L1, L2, and G1 was completely ossified but in G2 (the only juvenile bird, 7 weeks old), as well as K1, K2, and K4, there was a round nonossified area centrally (Figure 1). In K3, this area was still visible as a translucent area to the naked eye but when tapped was clearly calcified. Os ectethmoidale and Os lacrimale delimited the rostral extent of each Orbita. Os lacrimale articulated with Os frontalis, Os nasale (at Sutura lacrimonasalis) and Arcus jugale (at Articulatio jugolacrimalis) and, as has been observed by other authors, approached the latter bone more closely in Spheniscidae than in many other birds (Baumel et al., 1993). Flanking each Orbita ventrolaterally was the sigmoidal Arcus jugalis, a composite bone that is said to comprises part of Os maxillare, Os jugale and Os quadratojugalis. We found three separate skeletal elements to Arcus jugale on micro‐CT of L1 but a distinction could not be made between Os quadratojugale and Os jugale in either G1 or K1; in the case of the latter, the resolution may have been too low to allow such visualization but this was not the case in G1 (Figure 13). Neither could we separately identify Os jugale from Os quadratojugale when we disarticulated G2 (Figure 12). The bony Palatus, ventral to Orbitae, comprised paired plate‐like Ossa pterygoidea caudally and more rostral Ossa palatina, the two contacting at Articulatio pteryogopalatina. The central portion of Os palatinum, which articulated with the midline Rostrum parasphenoidale of Calvaria, was flattened dorsoventrally. From this slightly curved “plate” Processus pteygoideus extended caudally. There were two rostral processes, the long and slender Processus maxillaris, which articulated with Os maxillare and Os premaxillare and included the bending area of Zona flexoria palatina, and the more central and shorter Processus rostralis, which articulated with Vomer (Figure 12). The latter, a tall, thin, paired but fused bone, lay in the sagittal midline and in coronal section was slightly X‐shaped.

**Figure 14:**
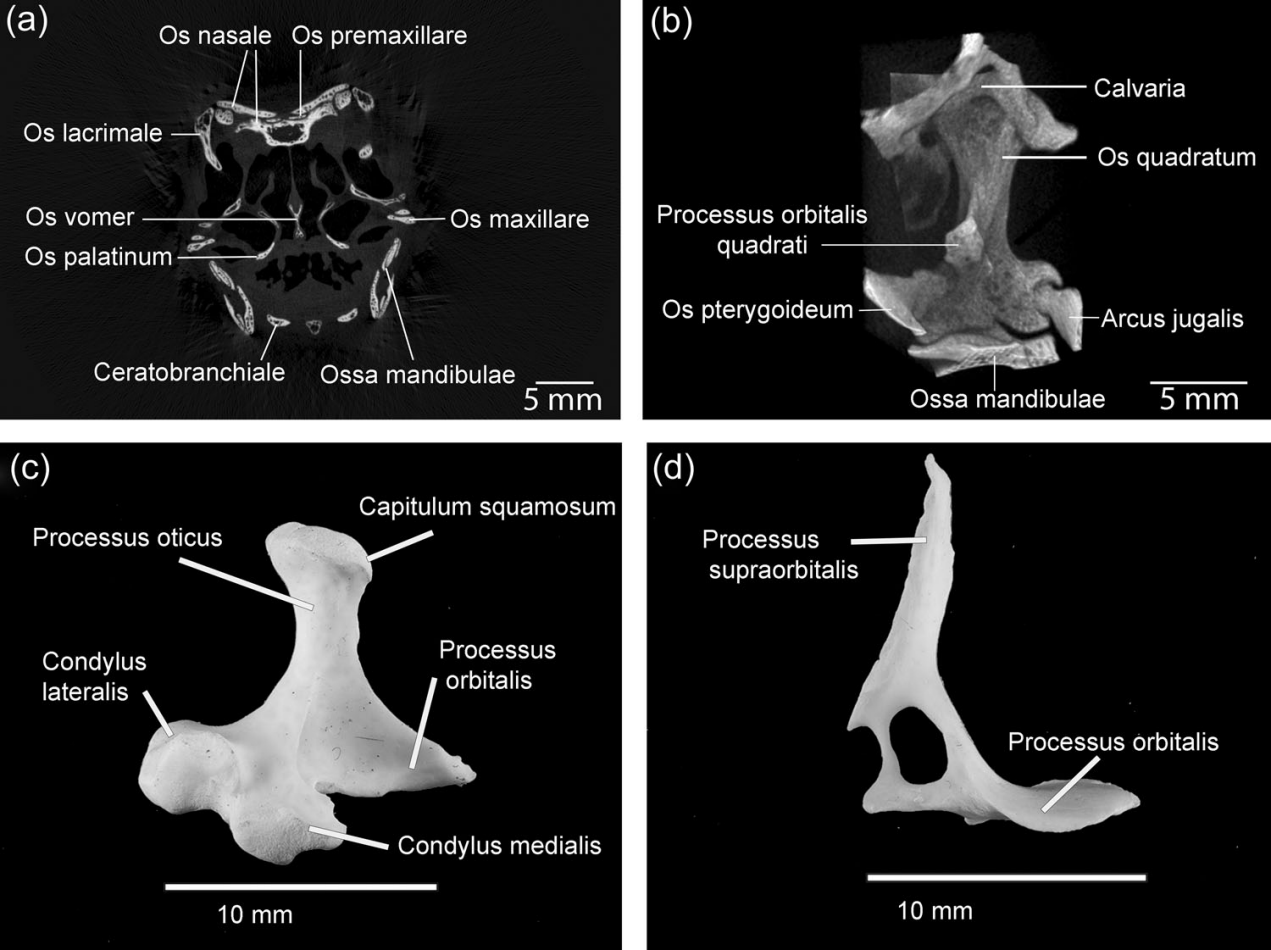
*Eudyptula minor* (specimen L1), digital reconstructions using CT Vox of small skeletal elements, demonstrating the resolution achievable with micro‐CT of frozen penguin heads versus magnified photographs. (a) Coronal section through the skull at the level of Os lacrimale. Clear separations were visible between the different bones of Maxilla, including between Os nasale and Os premaxillare, and between those of Ossa mandibulae. (b) Os quadratum sinistrum of L1, rostral aspect. The ball and socket synovial joints between Os quadratum and all the bones around it bar Ossa mandibulae were clearly visible. Little interlocking was visible at Articulatio quadratomandibularis between Os quadratum and Ossa mandibulae, although condyli medialis et lateralis as well as the less distinct Condylus caudalis were all visible. (c) Macrophotograph of Os quadratum sinistrum of L2, viewed from caudal, medial, and slightly ventral. (d) Medial view of Os lacrimale dextrum of L2, macrophotograph. The magnification afforded by a microscope clearly afforded a higher resolution than that obtained using micro‐CT. However, unlike with micro‐CT, the internal structure cannot be visualized in a nondestructive way nor can the joints with neighboring bones

**Figure 15:**
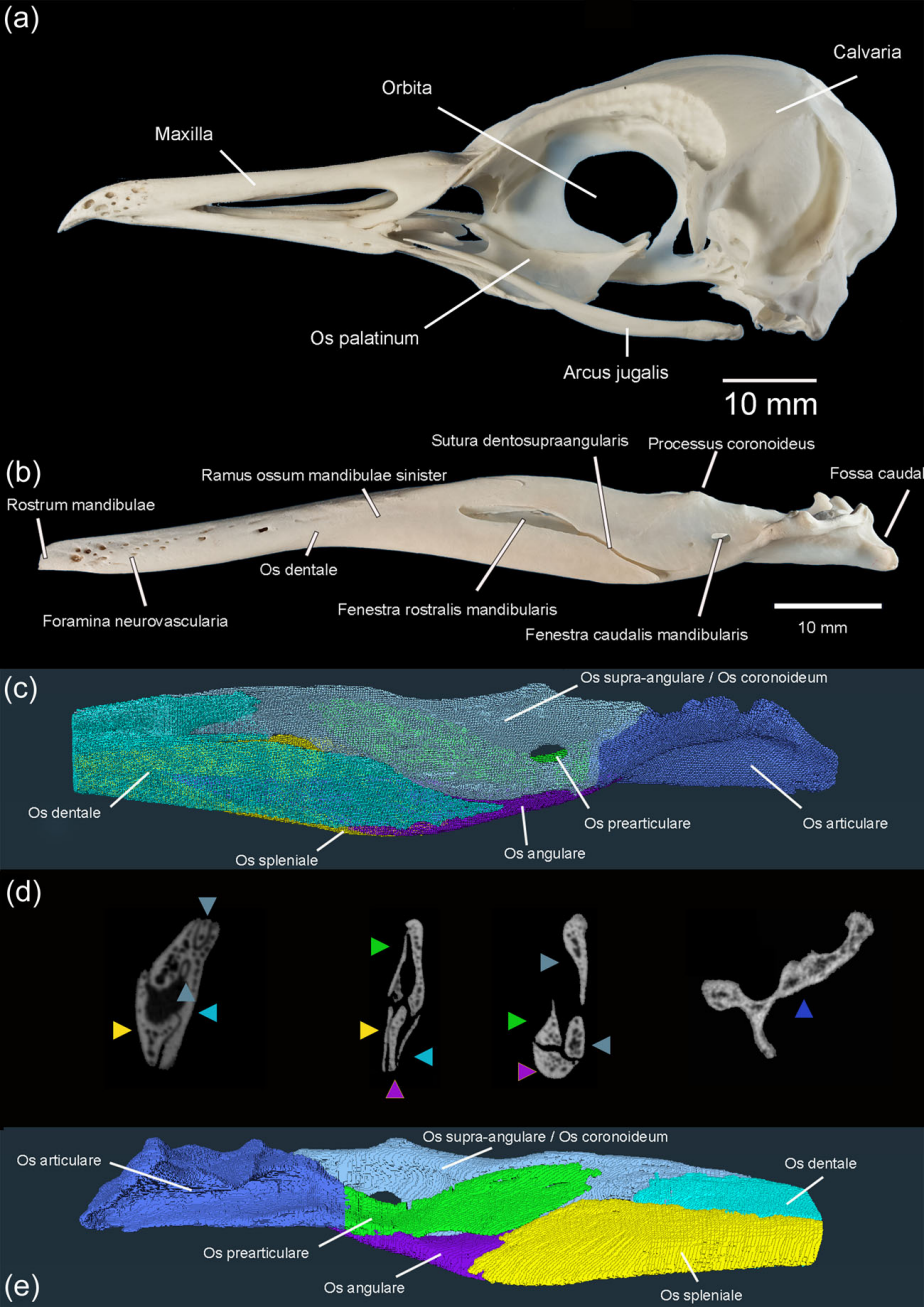
*Eudyptula minor*, macrophotographs and micro‐CT reconstructions of the large skeletal elements of Caput). (a) Macrophotograph of Caput (specimen L2), left lateral view. (b) Macrophotograph of Ossa mandibulae sinsitra, penguin L2, lateral view. Of the sutures, only Sutura dentosupraangularis is clearly visible. (c) Amira 2021.2 reconstruction of Ossa mandibulae sinistra, penguin L1, left lateral view, showing the individual skeletal elements within Ossa mandibulae. Rostral Ossa mandibulae were not imaged with micro‐CT and thus not reconstructed. (d) Coronal micro‐CT scans through Ossa mandibulae, (specimen L1), the rostro‐caudal position of each slice corresponding to its location under (c). Yellow arrowheads: Os spleniale. Light blue arrowheads: Os dentale. Gray arrowheads: Os supra‐angulare/Os coronoideum (unable to be separated). Green arrowheads: Os prearticulare. Purple arrowheads: Os angulare. Dark blue arrowheads: Os articulare. Note the two rostral processes of Os supra‐angulare. (e) Amira 2021.2 reconstruction of Ossa mandibulae sinistra, penguin L1, medial view, showing the individual skeletal elements within Ossa mandibulae

Ventrolaterally, Cranium articulated with Os quadratum via two condyles, both of which lay in the same joint cavity (Articulatio quadrato‐squamoso‐otica). This columnar bone had a rostral projection, Processus orbitalis, from which several Musculi mandibulae took origin. The ventral portion of Os quadratum articulated with Arcus jugalis at Articulatio quadrato‐quadratojugalis, Ossa mandibulae at the complex Articulatio quadratomandibularis (Figure 14) and, medially, Os pteryoideum (Articulatio quadrato‐pterygoidea). Ossa mandibulae are known consist of, in each of two rami, Os dentale, Os angulare, Os articulare, Os coronoideum, Os prearticulare, Os spleniale and Os supra‐angulare (Figure 15). However, on micro‐CT we were unable to separate Os supra‐angulare from Os coronoideum. There were two long processes that extended rostrally from Os supra‐angulare, one dorsal to and the other ventral to the dorsal portion of Os dentale. The two Rami mandibulae were fused at Rostrum mandibulae and were fenestrated rostrally in a similar manner to that seen in Os premaxillare. Each ramus extended caudally from Rostrum mandibulae at an angle of 8° in L1 (but 2° in K3 and G1) to the sagittal midline. At approximately halfway along each ramus there was a caudoventral bend and the angulation changed such that the angle each side made with the midline was 18° in L1, 11° in K3, and 12° in G1. Each also contained two oval fenestra, their long axes parallel to the edge of Ossa mandibulae, the rounder Fenestra caudalis mandibularis and the larger, more elongated Fenestra rostralis mandibularis. Prominent on the caudodorsal surface was Processus coroinodeus, to which attached the aponeurosis of Musculus adductor mandibulae externus pars rostralis. On micro‐CT, which did not image the most rostral portion of Ossa mandibulae, junctura were visible between the elements of Ossa mandibulae (Figure 14); Sutura dentosupraangularis between Os dentale and Os supra‐angulare was also visible to the naked eye. Lateral to Articulatio quadratomandibularis, Ossa mandibulae were attached via Ligamentum postorbitale to Processus postorbitale, while caudal to this hinge joint was the flattened Processus retroarticularis. An Articulatio mandibulosphenoidalis has been reported as present in some but not all Spheniscidae (Bock, 1960); the two bones remained 1.3 mm apart in L1 and 2 mm apart in K1 at the closest approach (imaging did not include this area in G1) and we could not detect an attachment on either dissection or diceCT.

**Figure 16:**
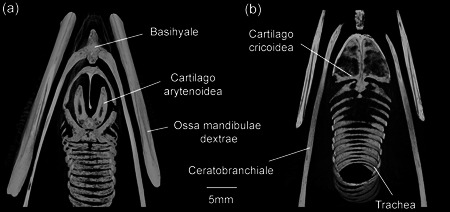
*Eudyptula minor*, CT Vox reconstructions of Larynx and Trachea using micro‐CT (specimen L1), demonstrating both the limit of resolution of skeletal elements and the ability of micro‐CT to retain accurate spatial orientation of these small bones, useful when drawing Figure 10. (a) Dorsal and (b) ventral view of Apparatus hyobranchialis. Ossification of Cartilago cricoidea was only just visible with very fine adjustment of the transfer function in CTVox for bone, because of the small degree of density differentiation from the surrounding tissue, a result of its thinness and depth. In particular, the parasagittal Cartilago cricoidea did not appear completely ossified when reconstructed despite raw data and dissection showing that it was. It was also observed that the first two tracheal rings were open dorsally and did not form a complete ring. They were also fused to each other, except where they articulated with the caudal extension of Cartilago cricoidea. The first five tracheal rings were all open ventrally. The more caudal rings were completely closed, although their ventral portion was thinner than the rest of the ring

The midline Basihyale was situated rostrally within Apparatus hyobranchialis and its two lateral processes articulated caudolaterally with the long straight Ceratobranchialia (Figure 16). Each Ceratobranchiale diverged at an angle of 15° to the midline in L1 and K1 (20° in G1) before articulating caudally with Epibranchiale, which curved dorsally as it headed further toward the caudal extremity of Caput. Articulating caudally with Basihyale was the midline Urohyale, which in L1 did not appear to be ossified on micro‐CT, while Larynx and, on its caudal margin, Trachea, was situated ventral to Apparatus hyobranchialis.

### 3.3 Soft tissue elements

**Figure 17:**
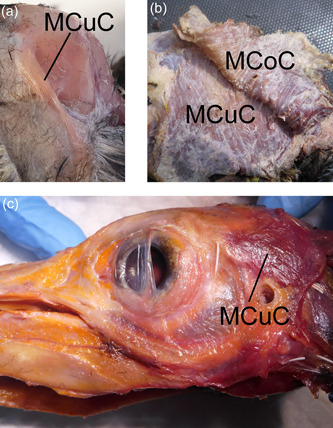
Left lateral view of Musculus cucullaris capitis (MCuC) in three species of penguin. There was a range of development of this structure from least to greatest in the little, gentoo and king penguin respectively. In Figure 7, we chose to illustrate little penguin (*Eudyptula minor*) L1, but clearly this penguin is not representative of all. (a) Musculus cucullaris capitis in little penguin L2. (b) Musculus cucullaris capitis in king penguin (*Aptenodytes patagonicus*) K1 was much larger than in L2 and extensive interdigitations with the overlying Musculus constrictor colli (MCoC) were apparent. (c) Musculus cucullaris capitis in gentoo penguin (*Pygoscelis papua*) G1

**Figure 18:**
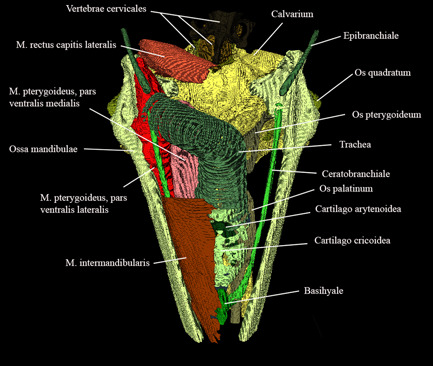
*Eudyptula minor*, Amira 2021.2 reconstruction of the head (specimen L1), ventral view, demonstrating the ability of diceCT to resolve the multiple anatomic elements of ventral Caput while ensuring accurate orientation of each with regard to other structures, a particular help in Figures 10 and 11. Overall length = 90 mm

**Figure 19:**
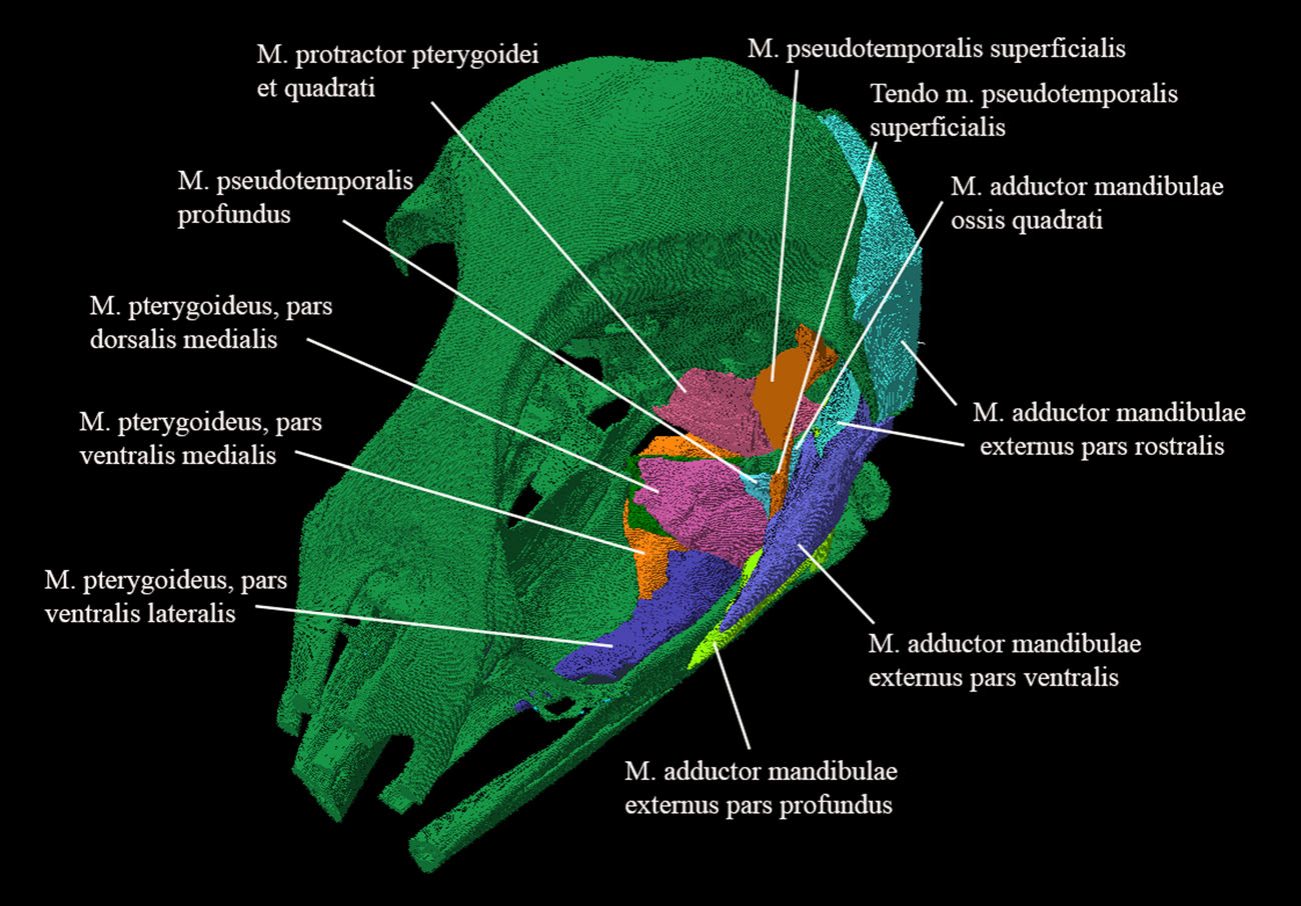
*Eudyptula minor*, Amira 2021.2 reconstruction of the head (specimen L1) from a left rostrodorsal view, demonstrating the ability of diceCT to resolve the complex but large musculi mandibulae, a three‐dimensional understanding of which was difficult with dissection alone given the destruction required to reveal deeper muscles. Overall length = 90 mm

**Figure 20:**
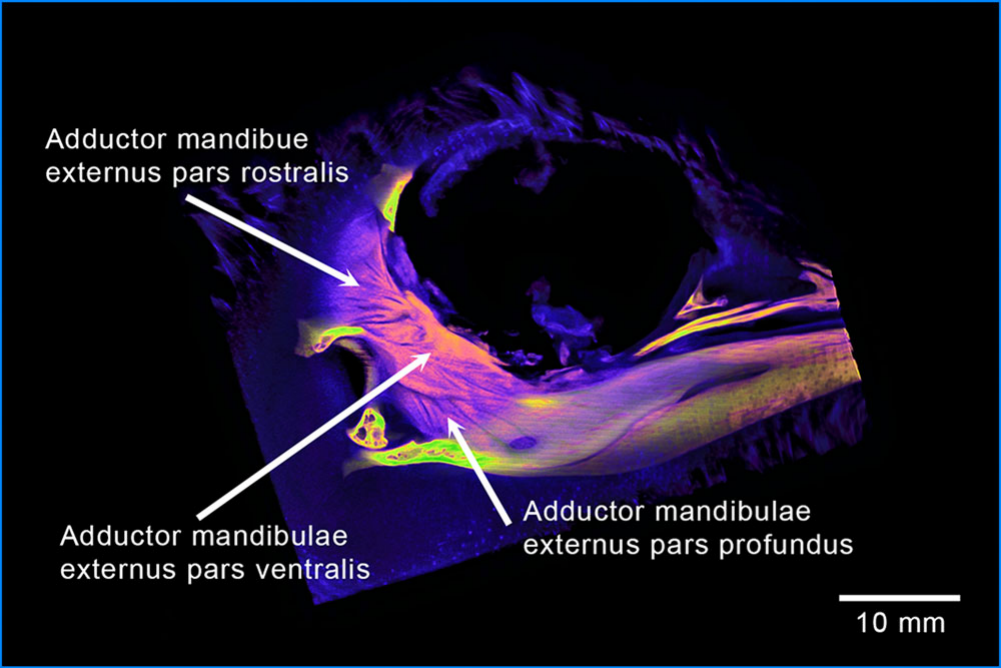
*Eudyptula minor*, CTVox reconstruction of the head (specimen L1), right lateral view, demonstrating the ability of diceCT to resolve the components and the orientation of the muscle fibers within each subdivision of Musculus adductor mandibulae externus dexter, which aided in the sketching of these details

**Figure 21:**
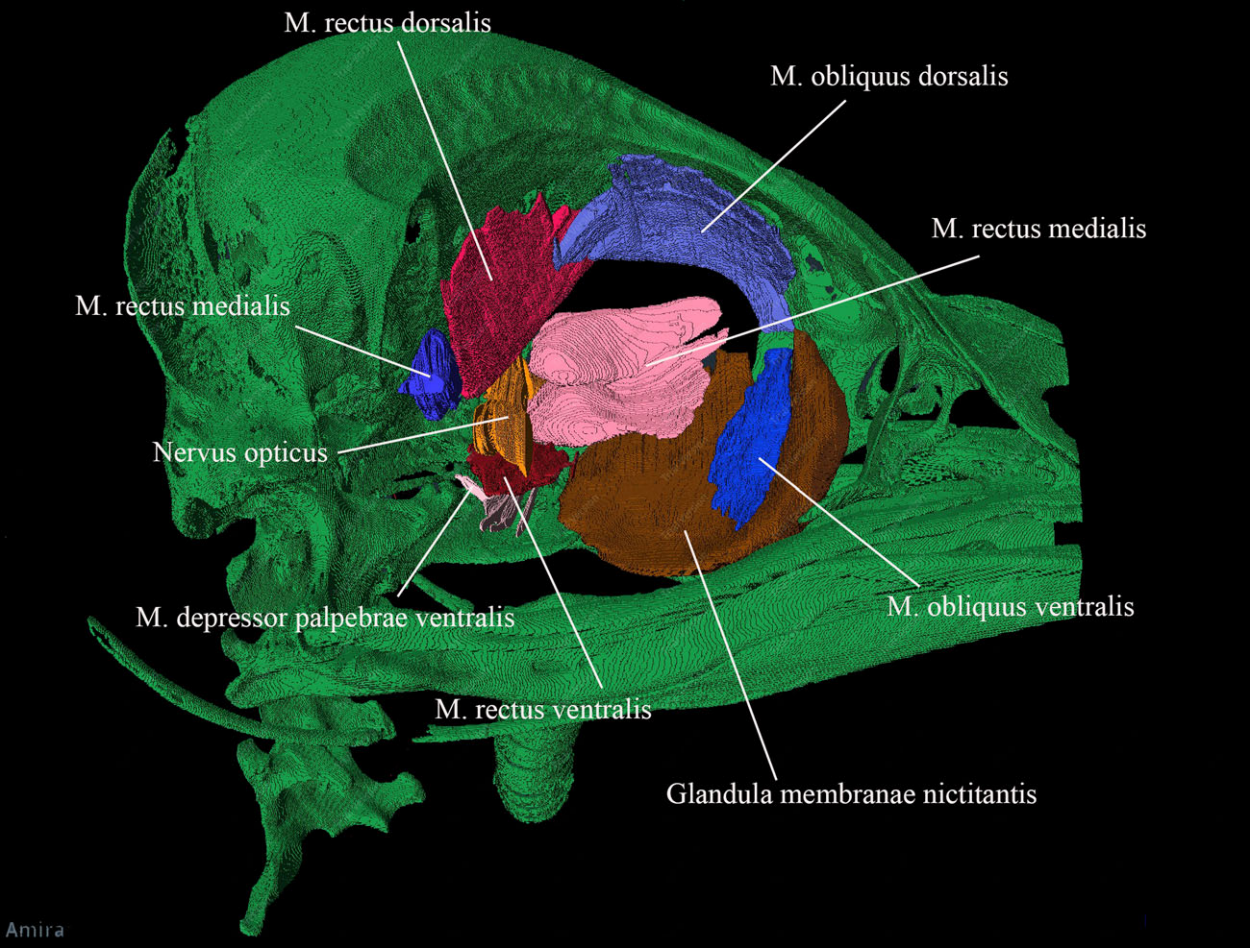
*Eudyptula minor*, Amira 2021.2 reconstruction of the head (specimen L1), right lateral view, demonstrating the ability of diceCT to view Musculi bulbi in relation to each other. This was a particular advantage when drawing Figure 8 (especially for Musculus obliquus dorsalis and Musculus obliquus ventralis), as during the process of dissection Bulbus oculi shrank considerably and the enucleation required to reveal these muscles disinserted them, distorting their anatomy considerably. On the other hand, the more distal portions of the muscles are thinner and unable to be automatically segmented, thus Figure 9A relied on dissection. Overall length = 90 mm

The origin, path, insertion, and general description of each muscle that we were able to positively identify is summarized in Table 3, together with the figures in which they feature. Notable was the variation in extent and thickness of Musculus cucullaris capitis even between different individuals of the same species (Figure 17). In L1, the animal we elected to illustrate, there were two separate divisions, both taking origin on Crista temporalis, one slightly more dorsal and deeper to the other. Very soon after its origin both split into multiple small bundles radiating both caudally and ventrally, those of the more dorsal division heading more caudally and those of the more ventral division heading more ventrally and interdigitating with similar bundles of fibers arising off the more superficial Musculus constrictor colli. G2 was similar to L1 in this regard. However, L2, G1, and K4 had only one muscle belly and Musculus cucullaris capitis was much larger, even adjusting for the relative size of the animal, in G1 than in either L1 or L2, and it was larger again in K2, such that it enveloped the entire back of the head. Amira and CTVox reconstructions of the areas of soft tissue of primary concern to this paper are shown in Figure 18, 19, 20, 21.

**Table 3:** Muscles mentioned in this paper, grouped according to anatomic location, together with the figures in which they appear

Muscle	Origin	Path	Description	Insertion	Figures references and notes
Musculi pterylarum (subcutanei)					
M. constrictor colli	Dorsal raphe	Circumferential around caudal Caput	Most superficial, thin sheet of muscle	Ventral raphe	Figures 6 and 17. Unable to be reconstructed using Amira
M. constrictor colli pars intermandibularis	Rostroventral M. contrisctor colli	Ventral and rostral	Fleshy band continuous caudally with M. constrictor colli	Ventral raphe	Figure 6. Unable to be reconstructed using Amira
M. cucullaris capitis	Crista temporalis	Ventral and caudal, deep to M. constrictor colli	Variable extent and either one or two divisions (see text)	Into fascia and with some connections M. constrictor colli	Figures 6, 7 and 17. Unable to be reconstructed using Amira
Musculi bulbi oculi					
M. rectus dorsalis	Periorbital tissue around Nervus opticus	Dorsal and rostral	Strap‐like muscle, 5 mm wide at insertion	Bulbus oculi, 8 mm posterior to limbus	Figures 8, 9, and 21
M. rectus ventralis	Periorbital tissue around Nervus opticus	Ventral and rostral	Strap‐like muscle, 5 mm wide at insertion	Bulbus oculi, 6.5 mm posterior to limbus	Figures 8, 9, and 21
M. rectus lateralis	Periorbital tissue around Nervus opticus	Lateral and slightly dorsal	Strap‐like muscle, 4.5 wide at insertion	Bulbus oculi, 5.5 mm posterior to limbus	Figures 8, 9, and 21
M. rectus medialis	Periorbital tissue around Nervus opticus	Rostral, bending laterally near insertion	Strap‐like muscle, at its widest 9 mm across and 1 mm thick; 5 mm wide at insertion	Bulbus oculi, in little penguin, 7.0 mm posterior to limbus	Figures 8, 9, and 21
M. obliquus dorsalis	Rostral Paries rostralis orbitae, approximately midway between dorsal and ventral and near the rim margin	Dorsal and caudal	Strap‐like, 3 mm across	Bulbus oculi, on rostral edge of M. rectus dorsalis insertion	Figures 8, 9, and 21
M. obliquus ventralis	Immediately ventral to M. obliquus dorsalis	Dorsal and caudal	Strap‐like, 3 mm across	Bulbus oculi, on rostral edge of M. rectus ventralis insertion	Figures 8, 9, and 21
M. quadratus membranae nictitantis	Much of the dorsal posterior surface of Bulbus oculi, from near Nervus opticus to 9.5 mm more anteriorly	Converges toward Nervus opticus	Thick muscle which lies flat against Bulbus oculi	Folds over itself to create a path (Vagina tendinis) through which Tendo m. pyramidalis passes	Figure 9
M. pyramidalis membranae nictitantis	Ventrally on Bulbus oculi	Becomes tendinous and heads rostrally through the Vagina tendinis created by M. quadratus membranae nictitantis	Smaller, triangular in shape with apex dorsal, toward its apex becomes tendinous	Membrana nictitans	Figure 9
M. levator palpebrae dorsalis	Rostrodorsal Margo orbitae	Passes caudoventral	Thin sheet‐like muscle	Palpebra dorsalis	Figure 6. Unable to be reconstructed with Amira
M. depressor palpebrae ventralis	Caudoventral Margo orbitae	Passes rostrodorsal	Thin sheet‐like muscle	Tarsus palpebrae ventralis	Figures 7 and 21
Musculi mandibulae					
M. adductor mandibulae externus pars rostralis	Fossa temporalis	Rostroventral	Fills Fossa temporalis, narrows rostrally as it passes between Processus zygomaticus and Processus postorbitalis	Via tendon to Processus coronoideus ossum mandibulae	Figures 6, 7, 19, and 20
M. adductor mandibulae externus pars ventralis	Two origins: Processus postorbitalis/Ligamentum postorbitale and Processus zygomaticus	Rostroventral	Sheet‐like somewhat triangular muscle	Wide insertion on caudolateral face of Ossa mandibulae	Figures 6, 7, 19, and 20
M. adductor mandibulae externus pars profunda	Os quadratum, corpus et processus oticus	Rostroventral	Sheet‐like muscle	Wide insertion Ossa mandibulae caudoventral to pars ventralis	Figures 6, 7, 19, and 20
M. adductor mandibulae ossis quadrati	Os quadratum, deep and rostral to M. adductor mandibulae externus pars profundus	Passes rostrally and ventrally, superficial to M. pseudotemporalis profundus	Strap‐like muscle	Ossa mandibulae, medial to Tuberculum pseudotemporale	Figures 7 and 20
M. pseudotemporalis superficialis	Os laterosphenoidale, medial to Processus postorbitalis	Ventral	Strap‐like muscle	Via tendon into Tuberculum pseudotemporale mandibulae	Figures 8, 19, and 23
M. pseudotemporalis profundus	Processus orbitalis quadrati, deep and rostral to M. adductor mandibulae ossis quadrati	Ventral and slightly rostral	Strap‐like muscle	Medial surface of Ossa mandibulae, rostral to M. pseudotemporalis superficialis	Figures 8, 19, and 23
M. pterygoideus pars ventralis lateralis	Lateral border of Os palatinum	Passes caudally, ventral to Os pterygoideum	Large strap‐like muscle, indistinguishable with pars ventralis medialis caudally.	Caudomedial Ossa mandibulae and Processus medialis mandibulae	Figures 11, 18, 19, and 23
M. pterygoideus pars ventralis medialis	Ventral surface of Os palatinum	Passes caudally, ventral to Os pterygoideum	Large strap‐like muscle, indistinguishable with pars ventralis medialis at insertion	Shares insertion with the above, but more medial fibers	Figures 11, 18, 19, and 23
M. pterygoideus pars dorsalis medialis	Dorsolateral Os palatinum	Passes laterally	Thin flat muscle	Medial aspect of Ossa mandibulae, ventral to the insertion of M. Pseudotemporalis profundus	Figures 11, 19, 23b, and 23c
M. protractor pteryoidei et quadrati	Paries caudalis orbitae	Passes laterally, ventrally and rostrally	Forms the majority of the nonbony portion of Paries caudalis orbitae	Deep surface of Processus opticus ossis quadrati and posterior edge of Os pterygoideum	Figures 11, 19, 23b, and 23c
M. depressor mandibulae	Ossa parietalia on either side of Prominentia cerebellaris	Ventral, passing superficial to Os exoccipitale	Processus postarticularis mandibulae	Strap like, with larger rostrolateral and smaller caudomedial parts (see text)	Figure 7
Musculi apparatus hyobranchialis					
M. intermandibularis (a.k.a. M. mylohyoideus)	Medial surface of Pars caudalis mandibulae	Ventral and medial	Two thin, flat (<1 mm thick) sheet‐like muscles, pars ventralis and dorsalis	Ventral midline raphe	Figures 6, 7, 18, and 24
M. serpihyoideus	Lateral aspect of Pars caudalis mandibulae, rostroventral to M. depressor mandibulae	Ventral, under urohyale. A medial invagination between Mm. intermandibularis and interceratobranchialis	A thin sheet of muscle	Ventral midline raphe	Figure 6. Unable to be reconstructed with Amira
M. stylohyoideus	Pars caudalis mandibulae, deep and ventral to M. serpihyoideus	Ventral, medial and rostral	Strap‐like muscle	Lateral aspect of Basihyale	Figure 10. Unable to be reconstructed with Amira
M. branchiomandibularis	Ventral margin of Ossa mandibulae, deep to M. intermandibularis and rostroventral to the insertion of M. pterygoideus pars ventralis lateralis	Complex course; passes caudoventrally deep to Mm. serpihyoideus and stylohyoideus	Strap‐like rostrally, wraps completely around Epibranchiale caudally	Wraps around Epibranchiale	Figure 10
M. interceratobranchialis	Midline raphe	Lateral and dorsal	Wide band‐like muscle	Caudomedial aspect of Ceratobranchiale	Figure 10. Unable to be reconstructed with Amira
M. ceratoglossus	Paraglossum	Caudal and slightly lateral	Small strap‐like muscle	Lateral aspect of Ceratobranchiale	Figure 10. Unable to be reconstructed with Amira
M. hyoglossus rostralis	Paraglossum	Directly caudal	Small, thin strap‐like muscle	Rostroventral Basihyale	Figure 10. Unable to be reconstructed with Amira
M. cricohyoideus ventralis	Ventral Os basihyale	Caudal and slightly lateral	Band‐like muscle, wider rostrally	Ventral Cartilago cricoidea	Figure 10. Unable to be reconstructed with Amira
M. tracheolateralis	Larynx	Caudal along the lateral side of Trachea	Long, thin band‐like muscle	Syrinx (not in our study)	Figure 10. Unable to be reconstructed with Amira
Musculi craniocervicales					
M. complexus	Vertebrales cercivales caudal to axis (not in our study)	Rostral, immediately beneath Musculi pterylarum	Large strap‐like muscle	Crista nuchalis transversa, close to the midline	Figure 7
M. splenius capitis	Dorsal surface of axis (not in our study)	Rostral, deep to M. complexus	Thick strap‐like muscle widens rostrally	Ventral to the insertion of M. complexus	Figure 8
M. rectus capitis lateralis	Rostral Vertebrae cervicales (not in our study)	Rostral and parallel to Vertebrae cervicales	Large muscle, oval in cross section	Ventrolateral Crista nuchalis transversa and caudal Processus paraoccipitalis	Figures 8, 11, and 18
M. rectus capitis dorsalis	Posterolateral surface of rostral Vertebrae cervicales (not in our study)	Rostral and parallel to Vertebrae cervicales	Large muscle, oval in cross section	Os basioccipitale	Figures 8 and 11
M. rectus capitis ventralis	Ventral surface of rostral Vertebrae cervicales (not in our study)	Rostral and parallel to Vertebrae cervicales	Large muscle, oval in cross section	Os basioccipitale, ventral to M. rectus capitis dorsalis	Figures 8 and 11

*Note*: Some muscles took origin caudal to Caput and thus were in part outside the range of our study, as noted. Muscles which could not be reconstructed using automated segmentation on Amira and for which we were therefore reliant on dissection are also noted.

Three glands were identified in Caput. Glandula membranae nictitantis (Figure 8, 9) was located deep to Musculus rectus ventralis and Musculus obliquus ventralis in the rostroventral part of Orbita, while Glandula lacrimalis occupied the caudodorsal portion of Orbita, caudal to Musculus rectus dorsalis (Figure 7, 8). Glandula nasalis was located in Fossa glandulae nasalis, as described previously (Figure 6). Bulbus oculi was visible in all penguins imaged with micro‐CT and was slightly oval, with an anteroposterior diameter of 14 mm in L1, 21 mm in G1 mm, and 28 mm in K1 and a transequatorial diameter of 19 mm in L1, 30 mm in G1, and 40 mm in K1.

## 4 DISCUSSION

As digital imaging becomes more widely available, studies that either incorporate or are solely reliant on such techniques are increasing. Digital morphology, however, may not always yield exactly the same result as traditional dissection. In this paper, both traditional techniques and digital reconstructions were used to obtain data and thereby inform illustration. Two questions arose from this process. First, is digital imaging better than classical dissection when attempting to understand anatomy? Second, do digital reconstructions depict anatomy better than traditional illustrations?

### 4.1 Digital imaging and reconstruction

The major advantages of micro‐CT are the ability to image in situ, the creation of a permanent data set that is not subject to future degradation and the potential for manipulation of the original data set at a later date. Of particular utility in this study, given the back‐and‐forth process of illustration, was the ability to repeatedly verify anatomic details. Volumetric analysis and measurement of spatial dimensions was also facilitated.

Using micro‐CT on frozen specimens eliminated potential bony distortion that may occur with any form of cleaning, a requirement of traditional anatomic methods (Hendry, 1999) and clearly present in the disarticulated Os premaxillare of G2 (Figure 12). It also maintained spatial orientation, ensuring accurate positioning of small bones or structures unattached to the skull, which might have become disarticulated or displaced if they were subject to dissection and cleaning, such as Apparatus hyobranchialis and Anulus ossicularis sclerae. Although a microscope could provide a more magnified view of the exterior of skeletal elements, micro‐CT could examine the internal structure of bones nondestructively, identify sutures between the small bones of Maxilla and Ossa mandibulae and display joint spaces, the correct three‐dimensional morphology of which were lost when bones were disarticulated (Figure 14). A limit was reached when attempting to reconstruct using CT Vox the entirety of thin and deep skeletal elements, such as Cartilago cricoidea, because of insufficient density differentiation from the tissue surrounding them (Figure 16), although there was sufficient contrast between bone and soft tissue on raw diceCT data to determine that the skeleton of the tongue, with the exception of Urohyale and Paraglossum, was ossified.

**Figure 22:**
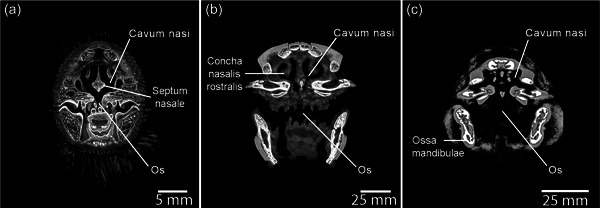
Coronal micro‐CT reconstructions using CT Vox of L1, G1, and K1 at corresponding points of Cavum nasi, demonstrating both the lack of distortion in these delicate structures that in situ imaging allows and the detail achievable, particularly in stained specimens, when imaging next to air‐filled cavities where density changes are large. (a) Stained head (diceCT) of kororā (*Eudyptula minor*) L1. (b) Unstained head of gentoo penguin (*Pygoscelis papua*) G1. (c) Unstained head of king penguin (*Aptenodytes patagonicus*) K1. More soft tissue details were visible in G1 and L1 than in K1, which was imaged using a lower resolution scanner. DiceCT added definition, especially to the mucosal surfaces of both Os and Cavum nasi, in L1. L1 had a much more arched Cavum nasi than did G1 while that of K1 was even flatter, perhaps a necessary result of the relative elongation of Maxilla in the latter

DiceCT required more specimen processing than did using frozen heads. Such preparation is known to cause tissue distortion and differential alteration of volumes (de Bournonville et al., 2019; Hedrick et al., 2018). However, it provided excellent visualization of soft tissues in situ. For instance, glandulae exocrinae were easily distinguished from muscles on diceCT due to a lesser affinity for iodine and CT Vox and Amira were both able to reconstruct subdivisions of complex large muscles, such as Musculus pterygoideus (Figure 18) and Musculus adductor mandibulae externus (Figures 19 and 20), as well as enable accurate orientation of the origins of Musculi bulbi (Figure 21). Thin tissues adjacent to air‐filled cavities, particularly Conchae cavi nasi, were particularly well imaged with micro‐CT. Many details of this area were visible even without staining, but the addition of contrast‐enhanced the mucosal surfaces and the outline of Conchae. The images obtained allowed a better appreciation of this complex structure than that obtained with the operating microscope, particularly for L1 and G1 although less so in the lower resolution images used for K1, most notably with regard to the ability to appreciate the three‐dimensional contours of these structures (Figure 22).

### 4.2 Classical dissection

**Figure 23:**
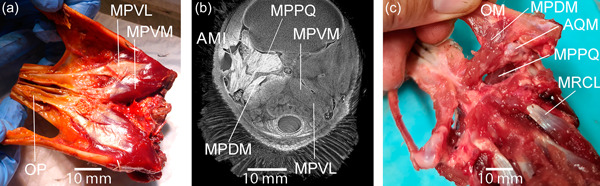
Despite their differences, good correlation could be seen between micro‐CT and anatomic dissection, even across genus boundaries, here with particular regard to Musculus pterygoideus, allowing both to be used in a complementary fashion when illustrating. (a) Ventral view of fresh dissection of gentoo penguin (*Pygoscelis papua*) G1 showing the two ventral divisions of Musculus pterygoideus (MPV), one lateral (MPVL) and one medial (MPVM). The latter inserts onto the posterior edge and the dorsal surface of os palatinum (OP). (b) Micro‐CT of little penguin (*Eudyptula minor*) L1, coronal section rostral to Musculus pterygoideus dorsalis medialis (MPDM). Staining was greater on the right side (left of the image), where the globe had been enucleated to allow better penetration of iodine. The origins and insertions of the muscles could be clearly identified, as could muscle fiber orientation. Musculus pterygoideus ventralis lateralis (MPVL) could be seen coming around onto the dorsal surface of Os palatinum. Muscles of the so‐called “Adductor mandibulae internus” (AMI) subsystem and Musculus protractor pterygoidei et quadrati (MPPQ) were also readily identifiable. (c) Fresh dissection of G2, dorsal view, demonstrating Musculus pterygoideus dorsalis medialis (MPDM). Articulatio quadratomandibularis (AQM) was opened and Ossa mandibulae (OM) everted in this image. Musculus rectus capitis lateralis (MRCL) was also visible in this view

Despite their differences, in general, there was a good correlation between diceCT images and dissections and the two were complementary (Figure 23). Further, there did not appear to be such major differences between fresh and fixed tissue as to impede informed illustration, our primary aim.

**Figure 24:**
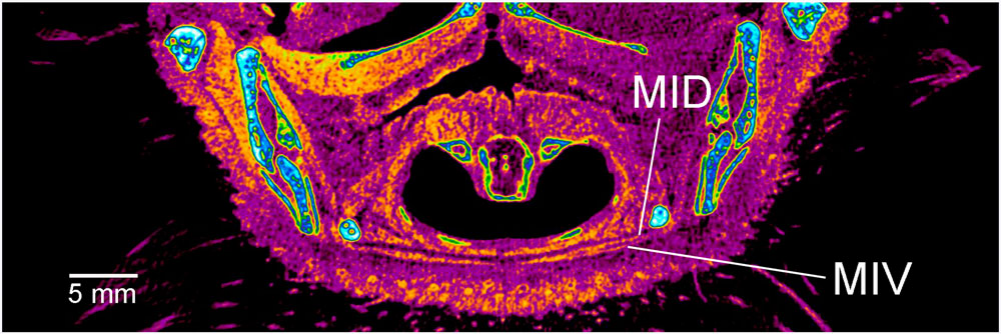
*Eudyptula minor*, coronal micro‐computed tomography (CT) section at the level of Larynx (specimen L1), demonstrating the lesser ability of micro‐CT to differentiate deeper, small soft tissue structures. Two separate divisions of Musculus intermandibularis, namely ventralis (MIV) and dorsalis (MID), were visible on the left of the skull (right of the image) but not the right, although on dissection with an operating microscope two were present throughout. Contrast between muscle and other soft tissues was better when they were adjacent to air‐filled spaces, including the external surface of the head and the empty right socket (left of the image), due to increased penetration of the iodine contrast material in these areas

**Figure 25:**
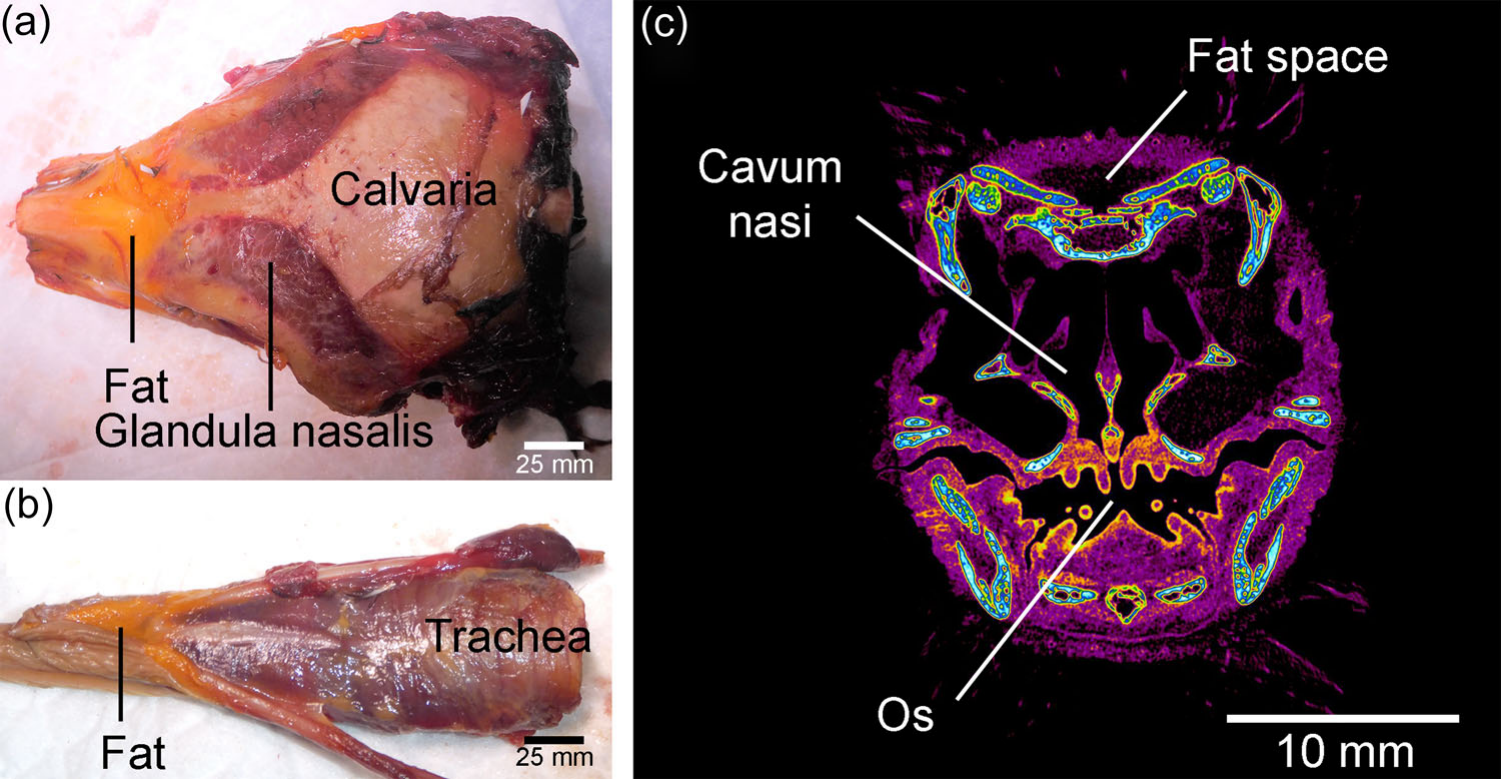
Fat bodies in Spheniscidae, visible on dissected specimens but removed by specimen preparation for diceCT and thus impossible to view on digital reconstructions. (a) Dorsal view of the skull of gentoo penguin (*Pygoscelis papua*) G1. A fat body was present in the depression above Nasus, between Calvaria and Maxilla. (b) Ventral view of Apparatus hyobranchialis and Trachea of G1. A fat body was visible ventral to the rostral part of Apparatus hyobranchialis. (c) Little penguin (*Eudyptula minor*) L1, coronal micro‐computed tomography section. The fat body dorsal to Nasus was removed by the process of staining, leaving a “space”

Dissection is cheap and does not require significant technology. It is thus more widely accessible than micro‐CT. Further, if available, multiple specimens may be dissected to answer questions that arise during the process of illustration without the time and expense that micro‐CT involves, and identify anatomic variation between animals, as was the case in regard to Musculus cucullaris capitis (Figure 17). The resolution afforded by dissection can also be increased by using magnification (Figure 14). In this study, PWH felt that traditional dissection more easily enabled accurate sketching of the external contours of muscles. It was also more accurate in the differentiation of the deep and smaller muscles of Apparatus hyobranchialis, as dissection using the operating microscope provided greater resolution than diceCT due to lesser density differentiation away from air‐filled spaces. For instance, diceCT images could only confirm on some sections that the little penguin has two divisions of Musculus intermandibularis (Figure 24), a muscle which is singular in some birds but dual in others (Baumel et al., 1993; McClearn & Noden, 1988) and a component part of Musculus constrictor ventralis (Holliday & Witmer, 2007). However, its dual nature was clearly apparent under the operating microscope and these two layers were easily able to be separated when dissecting L1. Automated Amira segmentation of Musculi pterylarum was also not possible and, although Musculus cucullaris capitis and Musculus constrictor colli were both identifiable on the raw micro‐CT scans, it would not have been possible to manually reconstructed them using Amira without almost totally reliance on the dissected specimen. Thus, they were drawn solely from dissection. This may be a problem not confined to this study, as we note that these muscles were not identified on a digital dissection of the rock dove (Jones et al., 2019). Fat bodies were also present on dissection, most prominently in gentoo G1, in the depression above the nose, along either side of the dorsal oral mucosa and below the rostral Apparatus hyobranchialis. However, the processing of L1 removed such deposits from the diceCT images, leaving an empty void (Figure 25). Finally, previous authors have come to contradictory conclusions regarding the patency of the external Nares gymnorhinales in *Eudyptula* (Pycraft, 1903; Zusi, 1975), of interest given its total occlusion and replacement by secondary external nares in the plunge‐diving northern gannet *Morus bassana* (McDonald, 1960). This inconsistency was also unable to be resolved using micro‐CT but simple irrigation of sinus antorbitalis with water showed that the Nares were patent in L2, G1, and G2.

On the other hand, dissection is a destructive technique and the same specimen cannot be dissected twice, although a photographic record can be made during the process. It also requires tissue manipulation, which can alter specimen interpretation. Dissection may be performed either on fresh tissue or formalin‐fixed tissue. Fixation allows for longer‐term preservation but, as with dice CT, causes more tissue alteration and is itself time‐consuming. The texture and color of tissues appear different in fresh versus fixed tissue. Further, when Integumentum is removed and the underlying soft tissues exposed to air, they dry out and contract away from each other, a process that in this study began within a matter of hours and further altered the morphology. In this study, we were unsure as to how distinct the two divisions of Musculus depressor mandibulae, as described by Sosa and Acosta Hospitaleche (2018) in adult Emperor penguins (*Aptenodytes forsteri*) and as we have illustrated, were. In particular, we wondered if our dissection accentuated a more minor distinction. When examining formalin‐fixed tissue, irrespective of the species it appeared that there were two divisions, although there was some interchange of muscle fibers. However, these two divisions were more difficult to discern on fresh tissue, especially in the ventral portion of the muscle. DiceCT was not able to differentiate two divisions, although this may have been because of insufficient resolution.

### 4.3 Digital reconstruction

A clear advantage of digital reconstruction over traditional illustration is the avoidance of the labor‐intensive process of the latter. Not only is an experienced artist required, but in all cases, the initial illustrations in this study had to be revised multiple times using both the actual specimen and micro‐CT images (minimum 2 and maximum 9) before they were considered accurate. Furthermore, no artist can possibly hope to make an illustration that reflects exactly the raw digital data in the way that a computer program can. However, some human input and interpretation is required even with digital renders. For instance, although automated digital segmentation using Amira is accurate for bone, where the contrast with other tissues is extremely high, segmentation of muscles requires an operator to trace their outline to obtain any useful image, which dissection can usefully inform. An awareness of potential errors introduced by perspective is also required, particularly when viewing two‐dimensional renders of three‐dimensional objects (Hadden et al., 2020).

### 4.4 Traditional illustration

Illustration and digital reconstruction should not be thought of mutually exclusive since the latter can inform the former, as was done in this study. Illustration possesses the ability to incorporate findings from both dissection and micro‐CT renders, an advantage that traditional illustration will always have over digital reconstructions, given that the latter, unless given specific manual instruction to the contrary, can only utilize digitally acquired raw data. The advent of micro‐CT can only serve to make illustration more accurate than if it were to rely on dissection alone, and thus more informative. An informed illustrator can also anticipate and compensate for changes that occur irrespective of specimen preparation and affect all forms of postmortem examination, such as the loss of volume in Bulbus oculi that occurs at death. As we hope the illustrations in this study demonstrate, a skilled artist can also, without changing the basic information presented, produce an illustration that is more aesthetically pleasing and more easily digested by the human brain than a digital reconstruction, and can emphasize notable features. This can be of particular importance to those new to the subject. Hence, just as landscape painting and portraiture has survived the advent of photography, so too we believe shall anatomic illustration.

### 4.5 Future considerations

In similar future studies, consideration could be given to leaving the specimen in iodine for longer to improve staining and allow deeper penetration (Gignac et al., 2016). However, there would likely still be differential staining between tissues closer to external surfaces and those deeper in such large specimens and thus require a compromise, although different, in the choice of scanning parameters. Greater resolution of individual structures would have been possible were we to have dissected out and imaged each element separately, as we have done previously for Anulus ossicularis sclerae (Hadden et al., 2021), although the relationships between structures would be lost. Perfusion‐based contrast enhancement using fresh specimens would be necessary were one to wish to image Systema Cardiovasculare. Finally, the illustration of a wider range of penguin species would further our understanding of the anatomic variation within Spheniscidae, as has been previously described for both extant and extinct species (Tambussi et al., 2015).

## 5 CONCLUSIONS

Micro‐CT scanning allows for detailed anatomic illustration, with frozen heads being useful for bony details and the avoidance of movement artefact, while fixation, dehydration, and staining allow for the differentiation of soft tissues and orientation of fibers within muscles. It is complementary to rather than a replacement for traditional dissection, just as digital reconstructions complement and inform illustration. Larger scanners can be used to image larger specimens, but such scanners have reduced resolving power and the longer X‐ray path length also reduces resolution. Penetration of iodine is limited by tissue volume, but the resolution of deeper structures can be improved by the removal of overlying tissue. It is likely that micro‐CT technology will continue to develop with time and that this will lead to improved resolution and research groups such as those that have led the development of dicect.com provide an expanding resource for others who wish to commence this type of study.

Detailed anatomic analysis of micro‐CT images may also offer new insights into penguin morphology although some aspects of the anatomy, such as the patency of the nares, may always be easier to ascertain using fresh tissue; a wider range of individuals than were in this study would be required to make definitive statements in this regard. Morphological differences correlate with diet and other behaviors and therefore are of use in determining trophic habits, including those of extinct penguins that one can no longer observe (Chávez‐Hoffmeister, 2020; Degrange et al., 2018; Haidr & Acosta Hospitaleche, 2014).

## AUTHOR CONTRIBUTIONS

**Peter W. Hadden**: Conceptualization (equal); data curation (supporting); formal analysis (equal); investigation (lead); methodology (equal); project administration (equal); validation (equal); visualization (equal); writing‐original draft (lead); writing–review and editing (lead). **William C. Ober**: Formal analysis (equal); methodology (equal); visualization (lead). **Dane A. Gerneke**: Data curation (lead); formal analysis (equal); methodology (equal); resources (equal); software (equal); visualization (equal); writing–review & editing (supporting). **Miriam Scadeng**: Data curation (supporting); formal analysis (equal); software (supporting); visualization (equal). **Charles N. J. McGhee**: Conceptualization (equal); project administration (equal); resources (lead); supervision (equal); writing–original draft (supporting); writing–review & editing (supporting). **Jie Zhang**: Conceptualization (equal); project administration (equal); resources (equal); supervision (lead); visualization (supporting); writing–original draft (supporting); writing–review & editing (supporting).

### TRANSPARENT PEER REVIEW

The peer review history for this article is available at https://publons.com/publon/10.1002/jmor.21476.

## Data Availability

The data that support the findings of this study, including micro‐CT stacks, photographs and the pupil video, together with renders made using this data, are available in the online open access repository https://figshare.com/search?q=10.17608/k6.auckland.c.5599341 (Digital Science, London, UK).
